# Long-Term Effectiveness of a Smartphone App for Improving Healthy Lifestyles in General Population in Primary Care: Randomized Controlled Trial (Evident II Study)

**DOI:** 10.2196/mhealth.9218

**Published:** 2018-04-27

**Authors:** Luis Garcia-Ortiz, Jose Ignacio Recio-Rodriguez, Cristina Agudo-Conde, María Carmen Patino-Alonso, Jose-Angel Maderuelo-Fernandez, Irene Repiso Gento, Elisa Puigdomenech Puig, Natividad Gonzalez-Viejo, Maria Soledad Arietaleanizbeaskoa, Yolanda Schmolling-Guinovart, Manuel Angel Gomez-Marcos, Emiliano Rodriguez-Sanchez, EVIDENT Investigators Group

**Affiliations:** ^1^ Primary Health Care Research Unit, Institute of Biomedical Research of Salamanca La Alamedilla Health Center Health Service of Castilla y León Salamanca Spain; ^2^ Department of Biomedical and Diagnostic Sciences University of Salamanca Salamanca Spain; ^3^ Department of Nurse and Physiotherapy University of Salamanca Salamanca Spain; ^4^ Department of Statistics University of Salamanca Salamanca Spain; ^5^ Valladolid Rural I Health Center, Health Service of Castilla y León Valladolid Spain; ^6^ Health Quality and Assessment Agency of Catalonia Barcelona Spain; ^7^ Torre Ramona Health Center, Health Service of Aragón Zaragoza Spain; ^8^ Primary Health Care Research Unit of Bizkaia, Basque Health Service-Osakidetza Bilbao Spain; ^9^ Río Tajo Health Center Health Service of Castilla-La Mancha University of Castilla-La Mancha Talavera de la Reina Spain; ^10^ Department of Medicine University of Salamanca Salamanca Spain; ^11^ Spanish Research Network for Preventive Activities and Health Promotion in Primary Care Salamanca Spain

**Keywords:** exercise, Mediterranean diet, smartphone, vascular stiffness

## Abstract

**Background:**

Information and communication technologies are currently among the supporting elements that may contribute to improving health and changing lifestyles.

**Objective:**

The aim of this study was to evaluate the long-term effectiveness of adding an app to standardized counseling in order to increase physical activity (PA) and adherence to the Mediterranean diet and to analyze the effects of app adherence in lifestyle changes.

**Methods:**

A randomized, multicenter clinical trial with a 12 month-follow up was conducted, involving 833 participants recruited by random sampling in 6 primary Spanish care centers (415 vs 418). Counseling on PA and the Mediterranean diet was given to both groups by a research nurse; however, the counseling + app group (intervention group) received additional training in the use of an app that was designed to promote the Mediterranean diet and PA over a 3-month period. Main outcomes and measures included PA by accelerometer and the 7-day Physical Activity Recall (PAR) questionnaire and adherence to the Mediterranean diet by an adherence screener questionnaire. We considered adherence to the app to be high when it was used for more than 60 days.

**Results:**

The mean age was 51 years (SD 12) in the intervention group and 52.3 years (SD 12.0) in the counseling-only group; females predominated in both groups (60.0%, 249/415 and 64.1%, 268/418, respectively). PA by accelerometer declined in both groups at 12 months (*P* value for tendency in moderate to vigorous PA, [MVPA]=.15). The intervention subgroup with high app adherence had better behavior than the low adherence subgroup (*P* value for tendency in MVPA=.001). PA analyzed by 7-day PAR did not show changes at 12 months in any of the groups (*P* value for tendency=.25). In the Mediterranean diet, an increase in adherence was observed in both groups at 12 months with no differences between them (*P* value for tendency=.46). In these two cases, the group with high app adherence also had better behavior, although without reaching significance for the tendency (*P*>.05).

**Conclusions:**

The participants with strongest app adherence showed better outcomes in terms of maintenance of healthy lifestyles at 12 months than those with weaker adherence. Overall, however, we found no differences between intervention group and counseling-only group in PA increase and adherence to the Mediterranean diet in the long term.

**Trial Registration:**

Clinicaltrials.gov NCT02016014; https://clinicaltrials.gov/ct2/show/NCT02016014 (Archived by WebCite at http://www.webcitation.org/6ymEXH6W4)

## Introduction

### Background

Noncommunicable diseases have been major public health concerns in recent years [1], and the prevention of such diseases is a major public health goal worldwide [2]. Lifestyle is one of the main causes of noncommunicable diseases, and the improvement in parameters such as dietary composition, physical activity (PA), and sedentary lifestyle are determinants for reducing the frequency of this type of pathology [3]. Information and communication technologies (ICTs) are currently used as support tools that can contribute to a change in the population’s lifestyle and thus improve their health [4].

A recent meta-analysis [5] found modest evidence for the efficacy of app interventions in dietary improvement, PA, and sedentary behaviors. The interventions that used an app in conjunction with other intervention strategies demonstrated improvements in behavioral and health outcomes compared with stand-alone app interventions [6]. These findings also showed that higher app usage was associated with improvements in PA and healthy eating [7], and low adherence rate was a major challenge in most of the studies, in particular, in studies with longer follow-up durations (>3 months) [8].

However, there are few studies that have analyzed the maintenance over time of the effect of an intervention with mobile apps once its use has ceased. Likewise, it does not seem to be clear to what extent greater or lesser adherence to the app affects the long-term maintenance of the beneficial lifestyle effects achieved during the time when the new technologies were being used. In the Evident II Project [9], an app was used for 3 months with the aim of achieving a change of lifestyle during this time with adherence to the Mediterranean diet (MD) and increased PA. We hoped that once acquired with the help of the app, the new habits would be maintained over time without the need for permanent reinforcement. This aspect does not seem to be sufficiently well clarified at present. In the analysis of short-term effectiveness, this study [10] showed that moderate -vigorous PA (MVPA) and adherence to MD increased from baseline in both groups, but no differences were found when comparing the increases between them. The evidence on ICT effectiveness was derived mainly from short-term (<6 months) experimental studies with far less data on long-term effectiveness or sustainability [8]. Therefore, few studies have examined effectiveness in large population samples and long-term follow-up studies using an app combining PA and dietary habits, and more evidence from studies with longer duration of follow-up periods seems necessary.

### Objectives

This study evaluated the long-term effectiveness of adding an app over 3 months to support standardized counseling in increasing PA and adherence to MD, and analyzed the effects of the time of app use on lifestyle modifications, as well as the maintenance over time of lifestyle changes achieved.

## Methods

### Design

The *Evident II* study was a randomized, controlled, multicenter clinical trial with 2 parallel groups. It was carried out with a follow-up period of 12-months [9] (Multimedia Appendix 1). Between January 2014 and September 2016, evaluations were made at baseline and after the completion of 3 and 12 months.

### Setting, Participants, and Randomization

**Figure 1:**
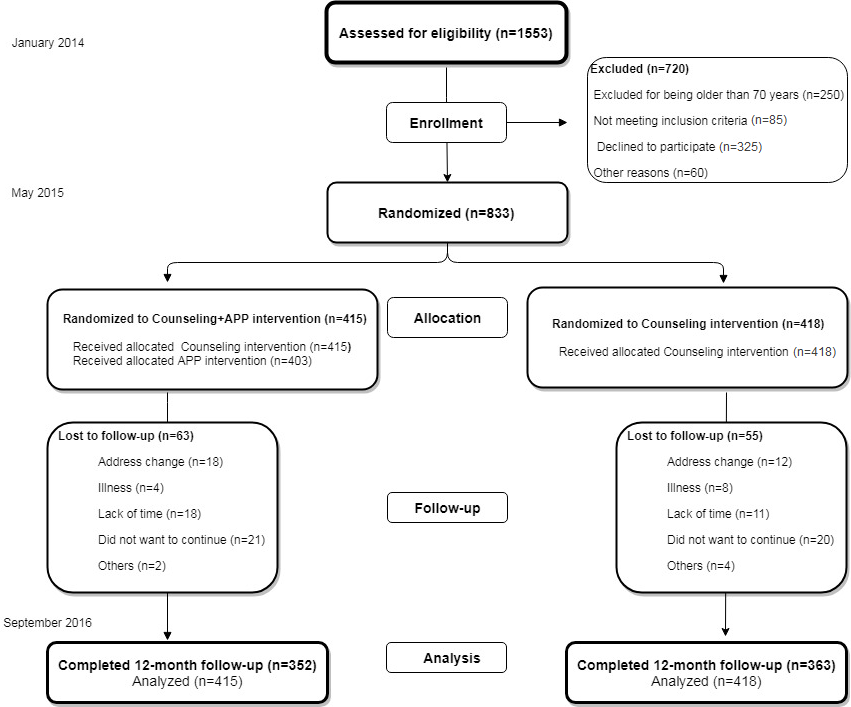
Flowchart of participants through of the Evident II trial comparing the counseling (intervention) + app group (IG) with the counseling-only group (CG).

The study population was selected from the Evident I study [11] using random sampling from 6 primary care centers. We excluded participants aged >70 years. A detailed description of inclusion and exclusion criteria has been published elsewhere [9]. The reasons for exclusion are summarized in Figure 1 (flowchart). Our study included 833 of the participants recruited in the Evident I study who were randomized in the ratio of 1:1 centrally from Salamanca using the Epidat 4.2 software package (Consellería de Sanidade, Xunta de Galicia,Spain) to assign them to the intervention group (IG, n=415) or the control group (CG, n=418). Due to the nature of the study, the participants could not be blinded to the intervention.

As reference committee, the Clinical Research Ethics Committee of the health care area of Salamanca approved the study (June 21, 2013), and the ethics committees of the 5 collaborating centers did likewise. All participants signed the informed consent form before inclusion in the study in accordance with the Declaration of Helsinki [12].

### Smartphone App for Evaluating Healthy Lifestyles

The smartphone app has a simple and user-friendly interface for daily recording of both portions of food eaten and PA performed (Multimedia Appendix 2) [9]. The app analyzed the user’s portion logs, applying standardized criteria to assess the quantity and quality of the food eaten each day and generated a detailed report on the composition of the diet and the calories consumed. A pedometer included in the smartphone app recorded the PA, and the subject could add PA information when it was not possible to use the pedometer (eg, when swimming). The app then generated a report on all the PA performed. After a daily analysis, the app generated a plan of recommendations for the following days with the aim of improving eating habits and increasing PA toward the targeted 10,000 steps per day. The information was stored in the device and downloaded on the day of the control visits for subsequent analysis.

### Intervention

The common intervention consisted of a 30-min standardized PA and MD counseling session, the effectiveness of which had previously been demonstrated [13,14]. It was performed in both groups by a trained research nurse, and participants received printed support material (leaflets) on the session. The participants in the IG group also received training from a different investigator in the use of a smartphone app. designed to promote MD and increase PA. An initial 15-min visit was used to provide training in the use of the device, which was then employed daily for the full 3-month intervention period. A detailed description of the intervention and app utilities has been published elsewhere [9]. A second visit took place 1 week after supplying the device in order to confirm that it was being used correctly. Participants returned the smartphone after 3 months, coinciding with the common follow-up visit.

### Outcome Measures and Follow-Up

The primary outcome measures were changes in PA, assessed by questionnaire and accelerometer, and adherence to MD, by questionnaire, at 12 months in IG compared with CG. Likewise, we analyzed PA changes and MD adherence in IG in relation to levels of app adherence. A detailed description of the method by which clinical data were collected has been published elsewhere [9].

### Physical Activity

The ActiGraph GT3X accelerometer (ActiGraph, Shalimar, FL, USA) [15] was used to objectively evaluate PA. For 7 consecutive days during routine PA, participants wore the accelerometer attached with an elastic strap to the right side of the waist except during bathing and performing water-related activities. The accelerometer was set to record PA data minute by minute. Inclusion criteria consisted of a minimum of 5 days of recordings, including at least 1 weekend day and at least 600 registered minutes per day. The first and last day data were excluded in order to analyze full days only, and the uptime was adjusted to 7 days. Activity “counts” were recorded to the internal memory of the accelerometers by converting acceleration units over a given period [16]. The main outcome variable from the activity monitor was the mean intensity of PA (counts per minute). The intensity of PA was rated according to the cutoff points proposed by Freedson [17].

PAR is a 7-day semistructured interview lasting 10 to 15 min in which participants provide an estimate of the number of hours dedicated to physical or occupational activities requiring at least moderate effort over the past 7 days [18,19]. The categories consisted of moderate, vigorous, and very vigorous PA. The dose of physical exercise was estimated in metabolic equivalent (MET) minutes per week.

### Nutrition

Adherence to MD as a nutrition primary endpoint was measured using the validated 14-point Mediterranean Diet Adherence Screener [20], developed by the PREDIMED study group. This 14-item screening questionnaire comprises 12 questions on food consumption frequency and 2 questions on food intake habits. Each question was scored as 0 or 1, and the total scores ranged from 0 to 14. A total score of ≥9 points represented adequate MD adherence.

### Adherence to the Smartphone App

In IG, adherence to the smartphone app was assessed through the number of recorded days in the device. We classified recordings into 4 categories, that were (1) 0 days, (2) 1 to 30 days, (3) 31 to 60 days, and (4) >60 days. A food log of >60 days in the 3 months that the participant had the smartphone represented high adherence, with ≤60 days equating to low adherence.

### Statistical Analysis

We estimated the sample size in relation to the main study endpoints. Assuming alpha=.05 and beta=.20 with an SD of 154 counts per min, in order to detect an increase of 30 counts per min in the IG versus CG groups we would have needed 828 participants (414 per group); for an SD of 2 points in MD, we would have needed 504 participants (252 per group) in order to detect an increase of 0.5 points. We considered 833 participants sufficient to detect clinically relevant differences in the main study endpoints.

The results were expressed as mean and SD for quantitative variables and as the frequency distribution for qualitative variables. Analysis of the results was made on an intention-to-treat basis. Chi-square and Fisher exact tests were used to analyze the association between independent qualitative variables. Student *t* test was used for the comparison of means between 2 groups and the paired *t* test or McNemar test was applied to assess changes within the same group. In order to analyze the effects of the interventions, we compared the changes observed between IG and CG by analyzing covariance while adjusting for each variable’s baseline measurements. To assess the effects of app adherence, we divided IG into high and low adherence categories according to specified criteria and performed the same analyses. In order to analyze the group effects with respect to MD adherence and PA increase, we performed a multivariate analysis of the variance of repeated measures adjusted by the baseline measure and analyzed the interaction of the main variables with the group (IG vs CG). Later, in order to evaluate the effects of app adherence, we performed the same analysis considering the day’s app use as dichotomous and continuous variable. We also performed a Spearman (rho, ρ) correlation between the number of days of app use and the difference between 12-month and baseline measurements of the main variables to analyze the association between these variables. We used the Johnson-Neyman technique [21] to attempt to determine specific cutoff points (ie, the point at which the number of days of app use was significantly different on the dependent variable, using the motivation phase as a moderating variable).

The contrast in hypotheses established alpha of .05. The data were analyzed using the SPSS version 23.0 (IBM Corp, Armonk, NY, USA).

## Results

### Baseline Characteristics of the Participants and Follow-Up

The flowchart (Figure 1) shows the subjects included in each group in addition to those who dropped out and the causes of this throughout the study. From a total of 118 subjects, 14.2% (118/833) dropped out, 15.2% (63/415) in the IG group and 13.2% (55/418) in the CG group. Table 1 describes the baseline characteristics of 833 participants.

### Changes in Physical Activity and Adherence to the Mediterranean Diet

**Figure 2:**
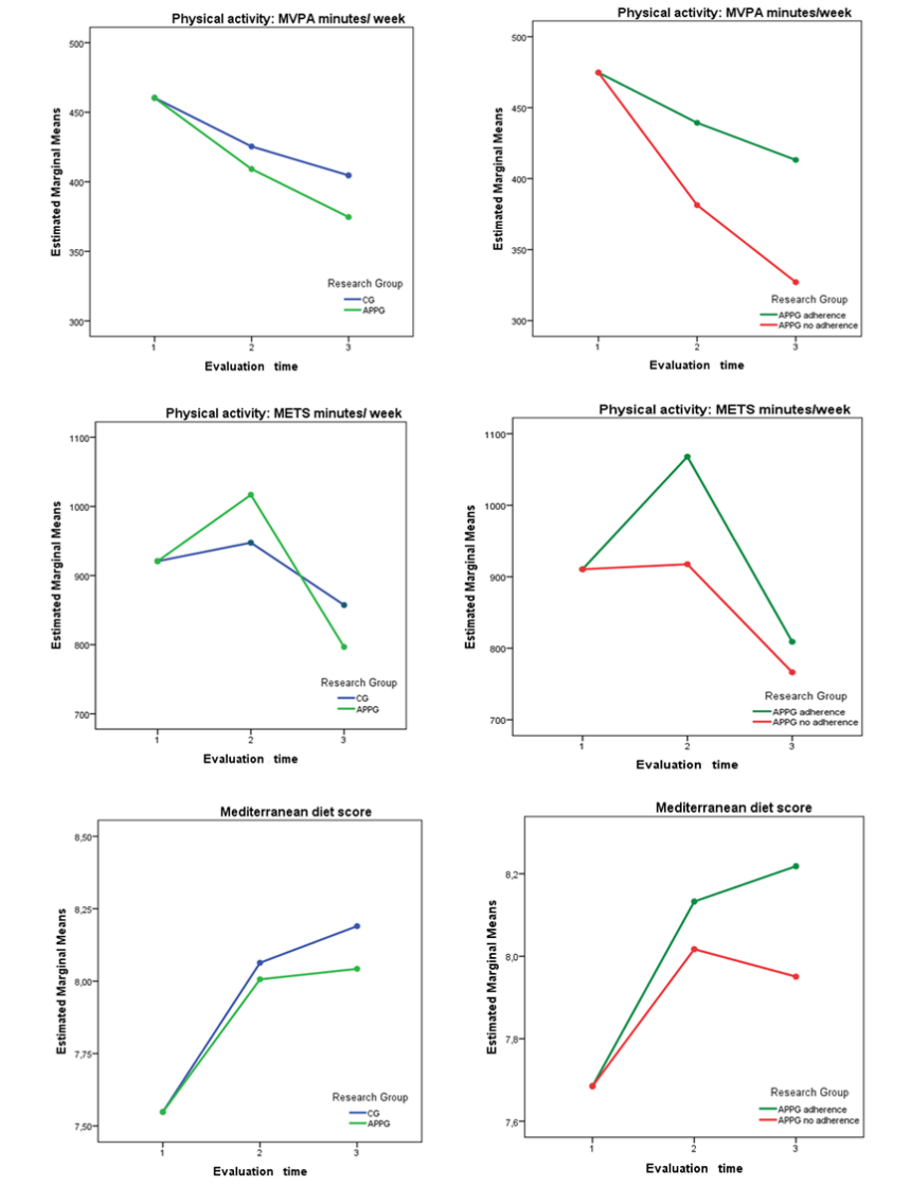
Evolution of physical activity and adherence to the Mediterranean diet at 3 and 12 months. (A) Comparison of the evolution of counseling + app group (APPG) versus the control group (CG) showing (1) moderate to vigorous physical activity (MVPA) minutes per week by accelerometer (P value for tendency=.15), (2) Metabolic equivalents (METs) minutes per week by 7-day Physical Activity Recall or PAR (P value for tendency=.245), and (3) Score of adherence to the Mediterranean diet (MD) (P value for tendency=.465). (B) Comparison of the evolution of APPG with high adherence versus IG with low adherence showing (1) MVPA min per week by accelerometer (P for tendency=.001), (2) METS min per week by 7-day PAR (P for tendency=.38), and (3) Score of adherence to the MD (P for tendency=.31). 1: Baseline, 2: Three months, 3: Twelve months.

In PA evaluated by accelerometer, there was a decrease in both groups with a greater decrease in IG without any interaction in the group effects (*P* value for tendency MVPA=.15), as you can see in Multimedia Appendix 3 and Figure 2. Regarding the 7-day PAR, no differences were found between the 2 groups in PA at 12 months (Table 2). Figure 2 shows METs per week, which were summary measures of the 7-day PAR at 12 months with no differences seen between them (*P* value for tendency MET min per week=.25). In MD adherence, a similar increase was observed in both groups without differences between them (*P* value for tendency MD score=.46; see Multimedia Appendix 4 and Figure 2).

### Adherence to the Smartphone App

**Figure 3:**
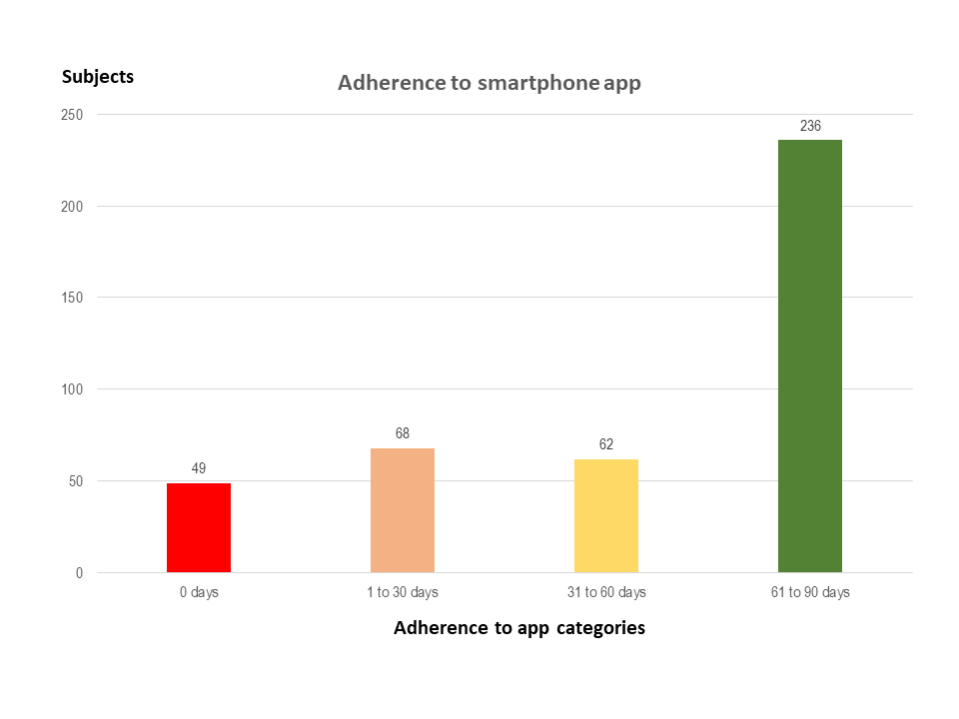
Adherence to the smartphone app (number of days with a record in the app). High adherence (>60 days)=57%; Low adherence (≤60 days)=43%.

The median use of the app was 67 days. Some participants (56.8%, 236/415) in the IG group had high app adherence, and 28.2% (117/415) of the participants used it for less than a month (Figure 3). The participants with low adherence were younger (49.5 vs 52.9 years), and there was a higher proportion of smokers (54.3%, 51/179 vs 45.7%, 43/236), as shown in Table 2.

We did not find a correlation between the number of days the app was used with the change at 12 months in the global score of the MD questionnaire, or with PA as measured with 7-day PAR. However, a correlation was found between the accelerometer changes in terms of steps per day (ρ=.114, *P*=.046), counts per minute (ρ=.126, *P*=.03), minutes of moderate activity (ρ=.112, *P*=.049), minutes of MVPA (ρ=.113, *P*=.047), and METS minutes per week (ρ=.120, *P*=.04). In the variance analysis of repeated measures, we found an interaction between the number of days of app use and MVPA with accelerometer (*P* value for tendency=.004), METS mins per week with 7-day PAR (*P* value for tendency=.03), and the MD adherence score (*P* value for tendency=.04).

**Table 1:** Baseline characteristics of the study population (N=833). Categorical variables are expressed as n (%) and continuous variables as mean (SD).

Variable		Counseling + app (n=415, 49.8%)	Counseling (n=418, 50.2%)
Age in years, mean (SD)		51.4 (12.1)	52.3 (12.0)
Gender (female), n (%)		249 (60.0)	268 (64.1)
**Work situation, n (%)**			
	Works outside home	228 (54.9)	203 (48.6)
	Homemaker	53 (12.8)	72 (17.2)
	Retired	77 (18.6)	89 (21.3)
	Student	10 (2.4)	8 (1.9)
	Unemployed	47 (11.3)	46 (11.0)
**Educational level, n (%)**			
	University studies	117 (28.2)	132 (31.6)
	Middle or High school	208 (50.1)	208 (49.8)
	Elementary school	90 (21.7)	78 (18.7)
**Smoking, n (%)**			
	Nonsmoker	190 (45.8)	166 (39.7)
	Smoker	94 (22.7)	108 (25.8)
	Former smoker	131 (31.6)	144 (34.4)
**Alcohol, n (%)**			
	Abstemious	86 (23.2)	88 (23.7)
	Low risk	200 (53.9)	209 (56.3)
	Moderate risk	46 (12.4)	34 (9.2)
	High risk	39 (10.5)	40 (10.8)
Body mass index (kg/m^2^), mean (SD)		28.1 (5.1)	27.6 (4.6)
Systolic blood pressure (mm Hg), mean (SD)		124 (16)	124 (17)
Diastolic blood pressure (mm Hg), mean (SD)		76 (10)	76 (10)
Total cholesterol (mg/dL), mean (SD)		202 (35)	206 (37)
Triglycerides (mg/dL), mean (SD)		112 (63)	107 (67)
Hypertension, n (%)		145 (34.9)	133 (31.8)
Dyslipidemia, n (%)		116 (28.2)	113 (27.3)
Diabetes, n (%)		32 (7.7)	30 (7.2)
Obesity, n (%)		126 (30.4)	114 (27.3)

**Table 2:** Baseline characteristics of counseling + app group by adherence to the app.

Variable		Adherence to app groups		*P* value
		Low adherence (n=179, 43%)	High adherence (n=236, 57%)	
Age (years), mean (SD)		49.5 (12.5)	52.9 (11.6)	.004
**Gender, n (%)**				
	Female	111 (44.6)	138 (55.4)	.47
	Male	68 (41.0)	98 (59.0)	
**Civil status, n (%)**				
	Single	33 (42.3)	45 (57.7)	.20
	Married	122 (41.6)	171 (58.4)	
	Separated	20 (62.5)	12 (37.5)	
	Widower	3 (30.0)	7 (70.0)	
	Others	1 (50.0)	1 (50.0)	
**Work situation, n (%)**				
	Works outside of home	104 (45.6)	124 (54.4)	.25
	Homemaker	20 (37.7)	33 (62.3)	
	Retired	26 (33.8)	51 (66.2)	
	Student	5 (50.0)	5 (50.0)	
	Unemployed	24 (51.1)	23 (48.9)	
**Educational level, n (%)**				
	University studies	48 (41.0)	69 (59.0)	.81
	Middle or High school	90 (43.3)	118 (56.7)	
	Elementary school	41 (45.6)	49 (54.4)	
**Smoker status, n (%)**				
	Nonsmoker	78 (41.1)	112 (58.9)	.04
	Current smoker	51 (54.3)	43 (45.7)	
	Ex-smoker	50 (38.2)	81 (61.8)	
**Alcohol, n (%)**				
	Abstemious	35 (40.7)	51 (59.3)	.33
	Low risk	84 (42.0)	116 (58.0)	
	Moderate risk	18 (39.1)	28 (60.9)	
	High Risk	22 (56.4)	17 (43.6)	
Body mass index (kg/m^2^), mean (SD)		28 (5)	28 (5)	.47
Systolic blood pressure (mm Hg), mean (SD)		122 (16)	125 (15)	.10
Diastolic blood pressure (mm Hg), mean (SD)		76 (10)	77 (10)	.10
Total cholesterol (mg/dL), mean (SD)		201 (35)	203 (35)	.44
Triglycerides (mg/dL), mean (SD)		114 (68)	111 (59)	.68
**Obesity, n (%)**				
	No	118 (40.8)	171 (59.2)	.15
	Yes	61 (48.4)	65 (51.6)	
**Hypertension, n (%)**				
	No	123 (45.6)	147 (62.3)	.17
	Yes	56 (38.6)	89 (37.7)	
**Dyslipidemia, n (%)**				
	No	133 (45.1)	162 (54.9)	.14
	Yes	43 (37.1)	73 (62.9)	
**Diabetes, n (%)**				
	No	161 (42.0)	222 (58.0)	.12
	Yes	18 (56.3)	14 (43.8)	

In Figure 2, we can see that the low app adherence participants had worse PA behavior according to the accelerometer (*P*<.001 for MVPA tendency). In Multimedia Appendix 5, more favorable behavior (*P*<.05) was observed among the high adherence participants in all parameters of the accelerometer except for light and vigorous or very vigorous PA. In the 7-day PAR, the high adherence participants were the ones who increased PA at 3 months but decreased at 12 months (*P*=.38 for tendency MET min per week). We found no differences in any of the 7-day PAR parameters between the high and low adherence participants (Multimedia Appendix 5).

Regarding MD (Multimedia Appendix 6), participants in the high adherence category and CG had a similar behavior, whereas participants in the low adherence category were worse and showed a decline after 3 months, although this was not statistically significant (*P*=.31 for tendency MD score). We observed the best behavior among the high adherence participants, although statistical significance was only achieved after higher fruit and lower commercial dessert consumption.

In relation to Prochasca and Diclemente’s stages of motivation for change, we found a greater number of days of app use among subjects during the preparation and maintenance stages for change in PA but not in eating habits (Multimedia Appendix 7). Likewise, in the multivariate analysis, we found that the intervention was more effective in the high app adherence group among subjects in the preparation (*P* value for tendency=.04) and maintenance (*P* value for tendency=.004) stages of PA measured by accelerometer and in the preparation stage (*P* value for tendency=.01) in the MD score. We did not find interaction between the number of days of app use and motivation stage in either PA (*P*=.91) or in adherence to MD (*P*=.19).

### Analysis by Subgroups

In the multivariate analysis of repeated measures, including at baseline, 3, and 12 months for MVPA, as well as MET minutes per week, and MD, we did not find interaction of the study group in any of the subgroups analyzed, which included gender, age groups, educational level, employment status, and obesity.

## Discussion

### Principal Findings

The Evident II study is one of the largest studies of its kind worldwide, with one of the longest follow-up periods; this made it possible to analyze the long-term effects of smartphone apps in terms of lifestyle improvements. The main findings included an intervention based on counseling plus the use of a smartphone app during 3 months that did not increase accelerometry-evaluated PA after 12 months versus CG. After 12 months, both groups showed a decrease, mainly in IG subjects with low adherence to the app. With regard to PA evaluated with 7-day PAR, IG showed an increase after 3 months but only among participants with strong app adherence. This was followed by a decrease after 12 months, reaching levels similar to those seen in the other 2 subgroups. This finding suggests that the app may have contributed to more PA during the time it was used, with loss of the effect after discontinuation of the app use. In relation to MD, both groups experienced improved compliance that continued to increase after 3 months except among low app adherence participants who showed a slight decrease after 3 months.

The use of an app requires the users to spend some time each day to enter information about their food habits. This may constitute a barrier, particularly for those who are less motivated to change their living habits, favoring discontinuation of app use. In this context, following an initial period, the users may become accustomed to the messages generated by the app, with a subsequent loss of their added value. On the other hand, the low adherence participants are younger and smoke more; this group shows less desire to acquire healthy habits.

This could explain the poorer behavior among participants with low app adherence. Belonging to IG, which received the smartphone with the app, may have resulted in less attention paid to standardized counseling and subsequent adherence to it. Discontinuation of device use may likewise have caused these individuals to disregard the habits recommended by the app. Nevertheless, these data are consistent with the observations from other studies in which no clear evidence of the efficacy of ICTs in improving healthy habits has been observed [5,8].

### Comparison With Prior Work

Of the 23 studies analyzed in the systematic review published by Schoeppe et al [5] on the efficacy of apps in improving lifestyle, smartphones were only seen to have a favorable impact on food habits in 5 studies and resulted in increased PA in 9 studies. The methodologies used vary greatly, and the best techniques for modifying habits through the use of ICTs have not been identified to date. The results also showed that increased app use seemed to be associated with better outcomes in terms of both greater PA and healthier food habits [7,22,23]. These findings are consistent with our own data regarding app adherence. On the other hand, multicomponent interventions have been found to exert a greater effect than interventions involving the isolated use of an app [5]. In the review carried out by Flores-Mateo et al [24], the app group showed a decrease in body mass index and a slight increase in PA, although without differences between the groups. Similarly, an important recent trial with a follow-up period of 24 months recorded no effects in terms of weight loss or increased PA with the use of smartbands [25]. Direito et al [26] observed a decrease in the 2 groups after 8 weeks when comparing 2 apps and evaluating the effects on PA using accelerometry. Similar results were obtained by Wang et al [23] after examining the effects of a wearable sensor and short message service likewise using accelerometry. These findings were consistent with those from our study in which we used accelerometry to analyze PA. The first week was probably overestimated as a result of the Hawthorne effect, which gradually faded at the 3- and 12-month evaluations.

Using questionnaires, Meher et al [27] evaluated PA and observed an increase in PA in IG versus CG after 8 weeks, although this effect was lost by week 20 [27]. Similar results have been obtained by Safran-Naimark et al [28] in a clinical trial involving an app to promote increased PA and diet quality in which the outcomes were evaluated by a questionnaire. The authors observed an increase in PA and diet quality in IG versus CG, particularly in the subgroup with greatest app adherence. These results are consistent with our own findings with regard to the 7-day PAR. In general, this self-reporting method for assessing interventions appears to offer better outcomes, particularly in the short term, than when the outcomes are evaluated by accelerometry [9,27,28].

There is little evidence supporting the effectiveness of apps in improving food habits [29]. Elbert et al [30] evaluated the effect of 2 app–based interventions on the consumption of fruit and vegetables. The authors only observed an increase in fruit consumption in the group that received audio messages. In this regard, Mummah et al [31] were able to increase vegetable consumption as a result of an app designed to increase both the amount and variety of intake. Finally, Gullian et al [7] observed an association between app adherence and increased vegetable and fruit juice consumption. This finding reinforces the results from our study, in which improved behavior was observed with reference to both diet and PA among the participants with the strongest app adherence.

### Limitations

Our study also has several limitations. The main findings of the study were based on self-reported information about adherence to both MD and PA. The nature of the intervention precludes blinding of the participants, and this could have influenced the results. Certain populations may have experienced difficulties using the app and consequently decided to leave the study. Finally, the recorded loss rate of 14.2% (118/833) may have biased the final sample study composition.

### Conclusions

The participants with the strongest app adherence showed better outcomes in terms of maintenance of healthy lifestyles at 12 months than those with weaker adherence, especially among subjects in the preparation and maintenance motivation phases. Nevertheless, in global terms, no differences were found between IG and CG in terms of increased PA and adherence to MD over long term. Further studies are needed to determine the optimum intervention time for facilitating adherence to the new technologies, in addition to identifying those population groups in which these interventions are more likely to be successful.

## Appendix

Multimedia Appendix 1 Study protocol Evident II.

Multimedia Appendix 2 Main screens of the app.

Multimedia Appendix 3 Changes in physical activity from baseline to 12 months. Groups: 1: Intervention; 2: Control. MET: metabolic equivalent; MVPA: moderate-to-vigorous physical activity. 7-day PAR: 7-day Physical Activity Recall.

Multimedia Appendix 4 Changes of adherence to Mediterranean diet from baseline to 12 months. Groups: 1: Intervention; 2: Control.

Multimedia Appendix 5 Changes in physical activity from baseline to 12 months of counseling + app group by adherence to app. Groups: 1: Adherent; 2: Not adherent. Analysis within group by t test pair and between groups by analysis of covariance (ANCOVA) test adjusted for baseline measures. High adherent group: use of app for >60 days. Low adherent group: use of app for ≤60 days. MET: metabolic equivalent; MVPA: moderate to vigorous physical activity. 7-day PAR: 7-day Physical Activity Recall.

Multimedia Appendix 6 Changes in Mediterranean diet to 12 months counseling + app group by adherence to app. Groups: 1: Adherent; 2: Not adherent. Analysis within groups by t test pair and Friedman test and between groups by analysis of covariance (ANCOVA) test adjusted for baseline measures. High adherent group: use of app for >60 days. Low adherent group: use of app for ≤60 days.

Multimedia Appendix 7 Prochasca and Diclemente's stages of motivation for change.

## Abbreviations

CG: control group
ICT: information and communication technology
IG: intervention group
MD: Mediterranean Diet
MET: metabolic equivalent
MVPA: moderate to vigorous physical activity
PA: physical activity
PAR: Physical Activity Recall
