# Supplementary material for: Long-Term Effectiveness of a Smartphone App for Improving Healthy Lifestyles in General Population in Primary Care: Randomized Controlled Trial (Evident II Study)
Source: JMIR Mhealth Uhealth. 2018 Apr 27;6(4):e107. doi: 10.2196/mhealth.9218 (PMC5948409; doi:10.2196/mhealth.9218)
Supplement: Multimedia Appendix 1 [file mhealth_v6i4e107_app1.pdf]

GOBIERNO DE ESPAÑA
MINISTERIO DE ECONOMÍA Y COMPETITIVIDAD
Instituto de Salud Carlos III

Subdirección General de Evaluación y Fomento de la Investigación

2013

CONVOCATORIA DE AYUDAS DE PROYECTOS DE INVESTIGACIÓN EN SALUD
MEMORIA DE SOLICITUD

Expediente Nº PI13/00618

TITULO: **Effectiveness of using a Smartphone application in improving healthy lifestyles. Randomized clinical trial (EVIDENT II).**

INVESTIGADOR/A PRINCIPAL: Luis Garcia Ortiz

TIPO DE PROYECTO: ○ INDIVIDUAL ○ COORDINADO ◉ MULTICÉNTRICO

NOMBRE DEL IP COORDINADOR:
(Cumplimentar sólo en caso de proyectos coordinados)
Luis Garcia Ortiz

DURACION: ☒ **3 AÑOS**

RESUMEN (Objetivos y Metodología del Proyecto) **(Máximo 250 palabras)**

Objectives: To develop and validate a mobile tool for use in Smartphone supports as a first step, and to evaluate the effect of adding this new tool of information and communication technologies (ICT), to a standardized intervention to increase adherence to nutritional recommendations of the Mediterranean diet and increase physical activity as primary endpoint. Analyze adherence and intervention results from a gender perspective. Methodology: Multicenter, randomized double-blind, clinical trial with two parallel group, aimed at assessing the effects of adding an TIC tool, developed for the Smartphone application (intervention), in support of behavioral and educational recommendations (control) in the increased physical activity and adaptation to the Mediterranean dietary pattern. Population: 1215 included subjects younger than 70 years from the project EVIDENT who agree to participate. Measurement and interventions: Physical activity will be assessed with the accelerometer and the 7-PAR day and adaptation to the Mediterranean diet with a questionnaire of adherence and a food frequency survey. It will also assess vascular structure and function, central blood pressure, Augmentation index, pulse rate and of carotid intima-media thickness. The counsel to adaptation the Mediterranean diet (based on the project PREDIMED) and exercise (based on PEPAF project), will be common to both groups. The intervention group will be added training and use of an application on a Smartphone designed to promote healthy eating and increased physical activity.

TITLE: Effectiveness of using a Smartphone application in improving healthy lifestyles. Randomized clinical trial (EVIDENT II).

ABSTRACT (Objectives and Methodology of the project)

Objetivos: Desarrollar y validar una aplicacion para la utilizacion en soportes Smartphone, como primera fase y evaluar el efecto de anadir esta herramienta de tecnologia de la informacion y la comunicacion (TIC), a una intervencion estandarizada para incrementar la adherencia a las recomendaciones nutricionales de la dieta mediterranea y para incrementar la actividad fisica, como objetivo principal del estudio. Analizar la adherencia y los resultados de la intervencion desde la perspectiva de genero. Metodología: Ensayo clinico, de dos grupos paralelos, multicentrico, a doble ciego y aleatorizado dirigido a valorar los efectos de anadir una herramienta TIC, aplicacion desarrollada para el Smartphone (intervencion), como apoyo a recomendaciones conductuales y educativas (control) en el incremento de actividad fisica y adaptacion de la alimentacion al patron de dieta mediterranea. Población: se incluiran 1215 sujetos, menores de 70 anos procedentes del proyecto EVIDENT que acepten participar. Mediciones e intervención: Se valorara el ejercicio con el acelerometro y el 7-day PAR y la adaptacion a la dieta mediterranea con un cuestionario de adherencia y una encuesta de frecuencia alimentaria. Se evaluara tambien la estructura y funcion vascular con la presion arterial central, Augmentation index, velocidad del pulso, Cardio Ankle Vascular Index y grosor de intima media de carotida. El consejo sobre adaptacion a la dieta mediterranea (basado en el proyecto PREDIMED) y sobre ejercicio (basado en el proyecto PEPAF), sera comun a ambos grupos. Al grupo de intervencion se le anadira el entrenamiento y utilizacion durante 3 meses de una aplicacion en un Smarthphone disenada para fomentar una alimentacion saludable y un incremento del ejercicio fisico

Expediente Nº PI13/00618

INVESTIGADOR/A PRINCIPAL: Luis Garcia Ortiz

MEMORIA DE SOLICITUD PROYECTO DE INVESTIGACIÓN EN SALUD
SECCIÓN ANTECEDENTES Y ESTADO ACTUAL DEL TEMA

Finalidad del proyecto, antecedentes y estado actual de los conocimientos científico-técnicos, grupos nacionales o internacionales que trabajan en la línea específica del proyecto o en líneas afines.
Citar las referencias en el apartado siguiente: Bibliografía más relevante. Máximo 3 páginas.

Benefits of physical exercise

The benefits of physical activity, both biological and psychological, have been abundantly backed up by scientific evidence [1]. While lower cardiorespiratory fitness is associated with an increased risk of cardiovascular disease and all causes of mortality, better physical fitness is linked to a reduced risk of mortality [2]. The results of a meta-analysis of the influence of physical activity on blood pressure found a mean decrease of 3.8 mmHg in systolic blood pressure and of 2.6 mmHg in the diastolic, with a reduction in normotensive individuals of 2.6 and 1.8 mmHg, and 7.4 and 5.8 mmHg in those with hypertension, respectively [3]. Aerobic exercise may attenuate arterial hardening, as observed in the Baltimore Longitudinal Study, where male athletes had pulse wave velocity (PWV), augmentation Index (AIx), and systolic blood pressure which was lower than sedentary individuals [4], although the relationship is not so clear among women. Improved carotid elasticity has also been observed among previously sedentary individuals who start a physical exercise program [5]. Aerobic exercise has been linked to slower progression of atherosclerosis among men [6]. The results from the first phase of EVIDENT [7] show an association between physical exercise and improved vascular structure and function. However, some aspects have not yet been clarified, such as the effect of endurance exercises, alone or combined with aerobic exercises, on vascular structure and function [8]. A link has been reported between sitting time (6 versus less than 3 hours a day) and mortality in women (RR 1.34, 95% CI 1.25-1.44) and men (RR 1.17, 95% CI 1.11-1.24). But the results are more relevant if sitting time (6 hours a day) is combined with low physical activity (<24.5 MET/hours/week) with RR of 1.94 (95% CI 1.70-2.20) for women and 1.48 (95% CI 1.33-1.65) for men, compared to those spending less time seated and more time being active. Associations were strongest with mortality from cardiovascular disease, but in addition these associations were independent of the level of physical activity [9]. The EVIDENT study found greater arterial stiffness related to hours spent watching television [10].

Interventions to increase physical exercise

Despite the benefits of physical activity being abundantly clear, the proportion of people that can claim to be active [11] is low in our environment. A sedentary lifestyle is prevalent among 60% of men and 74% of women in England [12], while the PEPAF study in Spain found 75% of subjects to be sedentary [13]. Different methods have been tried in promoting physical activity. A Cochrane review concludes that the data do not support the hypothesis that multi-component community interventions effectively increase levels of physical activity in the population [14]. A recent meta-analysis of trials in primary care concludes that the promotion of physical activity among sedentary patients increases the number of subjects that become active at 12 months (OR 1.42, 95% CI 1.17 to 1.73), although long-term effects have not been demonstrated [15]. The intervention carried out as part of the PEPAF study achieved an increase in physical activity over the controls of 18 mins/week [95% CI 6-31 mins/week]; with a 1.3 MET-hour/week increase [95% CI, 0.4-2.2]. The proportion of the population achieving the minimum recommended level of physical activity was 3.9% higher in the intervention group (1.2% -6.9%) than in the control group. There are few randomized controlled clinical trials that have assessed the impact of interventions on reducing daily sitting time, but an intervention based on promoting incidental walking while working (e.g., taking meetings while walking rather than sitting) has reduced daily sitting time by 21 minutes/day [16]. There is even less evidence for people with chronic pathologies, although a recent intervention involving people with type 2 diabetes achieved a reduction in daily sitting time of 23 minutes/day [17]. In a recent meta-analysis it was concluded that the effects of interventions are not sustained beyond 12 months [18].

Benefits of the Mediterranean diet

The Mediterranean diet is currently considered to be the most healthy dietary model. It is the traditional dietary pattern of countries such as Greece, southern Italy and Spain, the main characteristics of which are: a) high intake of cereals, pulses, nuts, fruits and vegetables; (b) use of olive oil for cooking and for dressing salads and vegetables, and as the main source of visible fat in the form of monounsaturated fatty acids; (c) moderate to high intake of fish, (d)

moderate to low intake of chicken and dairy products, (e) low consumption of red meat and meat products; f) moderate intake of alcohol, mainly in the form of red wine consumed with meals [19]. There is evidence that the Mediterranean diet could have a positive effect on endothelial function [20], and in lowering peripheral blood pressure [21]. A link has also been established to vascular structure as analyzed through the carotid intima-media thickness [22]. The Mediterranean diet has been associated with lower all-cause mortality in numerous cohort studies of older people [23] and, more specifically, with lower mortality from coronary heart disease [24].

Interventions to improve diet (Mediterranean diet, etc.)

Intervention studies involving the Mediterranean diet are scarce. A randomized clinical trial with a modified model of the Mediterranean diet versus a control diet, the Lyon Diet Heart Study [25], concluded that the Mediterranean diet is associated with marked reductions in cardiovascular mortality and the incidence of cardiovascular complications in patients suffering a myocardial infarction. However, to date, no randomized clinical trial has been conducted to assess whether the effects of the Mediterranean diet were superior to those of a low-fat diet in the primary prevention of coronary heart disease. The PREDIMED (prevention through the Mediterranean diet) study [21], a randomized clinical trial of dietary intervention, aims to discover whether a Mediterranean diet supplemented with virgin olive oil or nuts can prevent the occurrence of major cardiovascular complications in subjects at high vascular risk compared to a low-fat diet. In its initial phase, the PREDIMED study intervention achieved an increase of 1.4 points in the score measuring the adherence to the Mediterranean diet [26] (1.8 points on average), compared with an increase of 0.3 in the control group [27]. In the first year of follow-up, this led to a decrease in the prevalence of metabolic syndrome and its components and the incidence of diabetes mellitus [27,28]. This same study has recently shown that subjects in the intervention groups (Mediterranean diet) were able to reduce the incidence of major cardiovascular complications by 30% at 4 years of follow-up [29].

Analysis of motivation for change

To analyze motivation for change towards healthier lifestyles, Prochaska and DiClemente's transtheoretical model of change [30] is used, which describes the phases of motivation or stages of change that a person goes through. This model proposes that individuals go through five stages to modify behaviours: precontemplation, contemplation, determination, action and maintenance.

Measurement of arterial aging

Aging is associated with an increase in arterial stiffness, which can be measured with different clinical and biological instruments. The currently adopted gold standard for measuring arterial stiffness is carotid-femoral pulse wave velocity (PWV) [31], which has been associated with increased morbidity and mortality in subjects with cardiovascular disease as well as healthy subjects [32, 33]. A closer association of central and peripheral blood pressure with cardiovascular morbidity and mortality has also been reported [34]. The central and peripheral augmentation index are also markers of arterial stiffness, which together with aortic systolic blood pressure and aortic pulse pressure complement the information obtained with pulse wave velocity. In the CAFÉ sub-study [32], it was observed that subjects who presented higher morbidity and mortality had a central arterial pressure and an augmentation index which was higher than the other group.

Information technologies in supporting improved health

Given the overwhelming trend in recent years towards the use of mobile phones, and recently with the introduction of smartphones, it may be estimated that in 10 years 80-90% of the population of developed countries will have one of these devices [35] and that they will probably be equipped with many more utilities than those currently in use. For information technologies, the area of □□health and medicine is proving to be an important field of application; for example, the usefulness of SMS in helping to quit smoking has recently been demonstrated [36]. The value of smartphone applications for use by health personnel, both in training and in decision support in routine clinical care and in emergency medicine has been increasing and there is already some evidence proving its utility [37]. They have also been used in patient care for the monitoring of biological parameters [38], detection of falls among elderly patients [39], prevention of cognitive impairment [40], diabetes monitoring [41], cardiac rehabilitation [42] and also in promoting physical activity [43] and the management of obesity [44]. The potential of mobile communications, especially among the smartphone generation, to transform health care and clinical interventions in the community is great [35], but, as recommended by the FDA, its effectiveness in multicentre clinical trials needs to be evaluated in a manner similar to drugs. A meta-analysis of the use of information technologies for dietary assessment concludes that they could improve dietary assessment among some population groups but need to improve the validity and reliability of micronutrient assessment [45]. The number of applications that have been developed in the field of health and

specifically food and exercise, and aimed at the general population, is great. Although acceptance and ease of use in terms of measuring food intake and physical activity have been assessed among young adults [46], it has not been tested with other age and population groups, nor has its effectiveness in relation to health outcomes. Not many studies have validated these tools, although there are some currently in progress [47]. Fewer still assess the impact that they can have on increasing physical activity and the improvement of the dietary patterns, and therefore the health, of those who use them [48].

This study aims to provide evidence of the effect of new information and communication technologies (ICTs), and specifically smartphone applications, as tools to support the change to healthy lifestyles. It will also evaluate the effects of these changes on arterial aging. Therefore, the purpose of this study is, firstly, to develop and validate an application for smartphones in order to support the standardized advice for increased physical activity and to make it easier to adapt eating habits to the recommendations of the Mediterranean diet. Secondly, it is the main objective of the project to quantify the effect that can be attributed to the application that has been developed on habit change. Thirdly, the study aims to evaluate the effect of habit change on vascular structure and function, and therefore on arterial aging.

This project continues the research focus of the project "Physical exercise, fitness and dietary pattern and their relationship with circadian blood pressure pattern, augmentation index and biological markers of endothelial dysfunction" (EVIDENT study), which is the precursor to the current study [7]. The main findings of EVIDENT I were that physical exercise is associated with more favourable circadian patterns, lower arterial stiffness and a lower cardiovascular age. We also found that a sedentary lifestyle (time watching television) is associated with increased arterial stiffness. Similarly, the intake of fat-rich dairy products is linked to less favourable vascular structure and function. Finally, we found an association of physical activity with inflammatory markers such as fibrinogen (data published or in press).

COORDINATION AND PARTICIPANTS: The project will be multicentre, coordinated by the Alamedilla Health Centre in Salamanca, and will involve the following centres:

Participantes centers: IP:

Centro de Salud La Alamedilla de Salamanca: Luis Garcia Ortiz (Coordinador)
Centro de Salud Casa del Barco de Valladolid: Amparo Gomez Arranz
Centro de Salud Algorta de Bizkaia: Eguskine Iturregui San Nicolas
Centro de Salud Torreramona de Zaragoza: Natividad Gonzalez Viejo
Centro de Salud Rio Tajo de Talavera de la Reina (Toledo): Yolanda Schmolling Guinovart
Centro de Salud Passeig de Sant Joan de Barcelona: Carlos Martin Cantera (financiado en 2012)

Expediente Nº PI13/00618

INVESTIGADOR/A PRINCIPAL: Luis Garcia Ortiz

MEMORIA DE SOLICITUD PROYECTO DE INVESTIGACIÓN EN SALUD
SECCIÓN ANTECEDENTES Y ESTADO ACTUAL DEL TEMA

Citar las referencias incluidas en el apartado anterior: Antecedentes y Estado actual. Máximo 1 página.

1.Varo Cenarruzabeitia JJ *et al*: *Med Clin (Barc)* 2003, 121(17):665-672.
2.Myers J *et al*: *N Engl J Med* 2002, 346(11):793-801.
3.Whelton SP *et al*: *Ann Intern Med* 2002, 136(7):493-503.
4.Vaitkevicius PV *et al*: *Circulation* 1993, 88(4 Pt 1):1456-1462.
5.Tanaka H *et al*: *Circulation* 2000, 102(11):1270-1275.
6.Lakka TA *et al*: *Ann Intern Med* 2001, 134(1):12-20.
7.Garcia-Ortiz L *et al*: *BMC Public Health* 2010, 10:233.
8.Seals DR *et al*: *J Appl Physiol* 2008, 105(4):1323-1332.
9.Patel AV *et al*: *Am J Epidemiol* 2010, 172(4):419-429.
10.Recio-Rodriguez JI *et al*: *Am J Hypertens* 2013, 26(4):488-494.
11.Haskell WL *et al*: *Med Sci Sports Exerc* 2007, 39(8):1423-1434.
12.Foster C *et al*: *Cochrane Database of Systematic Reviews* 2005(1).
13.Grandes G *et al*: *Arch Intern Med* 2009, 169(7):694-701.
14.Baker Philip RA *et al*: *Cochrane Database of Systematic Reviews* 2011(4).
15.Orrow G *et al*: *BMJ* 2012, 344:e1389.
16.Gilson ND *et al*: *Int J Behav Nutr Phys Act* 2009, 6:43.
17.De Greef KP *et al*: *Patient Educ Couns* 2011, 84(2):275-279.
18.Hobbs N *et al*: *BMC Med* 2013, 11(1):75.
19.Martinez-Gonzalez MA *et al*: *Eur J Epidemiol* 2004, 19(1):9-13.
20.Fuentes F *et al*: *Br J Nutr* 2008, 100(1):159-165.
21.Estruch R *et al*: *Ann Intern Med* 2006, 145(1):1-11.
22.Murie-Fernandez M *et al*: *Atherosclerosis* 2011, 219(1):158-162.
23.Trichopoulou A *et al*: *BMJ* 1995, 311(7018):1457-1460.
24.Martinez-Gonzalez MA *et al*: *Eur J Nutr* 2002, 41(4):153-160.
25.de Lorgeril M *et al*: *Circulation* 1999, 99(6):779-785.
26.Schroder H *et al*: *J Nutr* 2011, 141(6):1140-1145.
27.Salas-Salvado J *et al*: *Arch Intern Med* 2008, 168(22):2449-2458.
28.Salas-Salvado J *et al*: *Diabetes Care* 2011, 34(1):14-19.
29.Estruch R *et al*: *N Engl J Med* 2013.
30.Tuah NA *et al*: *Cochrane Database Syst Rev* 2011(10):CD008066.
31.Laurent S *et al*: *Eur Heart J* 2006, 27(21):2588-2605.
32.Williams B *et al*: *Circulation* 2006, 113(9):1213-1225.
33.Mattace-Raso FU *et al*: *Circulation* 2006, 113(5):657-663.
34.Roman MJ *et al*: *Hypertension* 2007, 50(1):197-203.
35.Boulos MN *et al*: *Biomed Eng Online* 2011, 10:24.
36.Free C *et al*: *Lancet* 2011, 378(9785):49-55.
37.Low D *et al*: *Anaesthesia* 2011, 66(4):255-262.
38.Neubert S *et al*: *Telemed J E Health* 2010, 16(4):504-509.
39.Yamada M *et al*: *Age Ageing* 2011, 40(4):516-519.
40.Dufau S *et al*: *PLoS One* 2011, 6(9):e24974.
41.Ciemins E *et al*: *J Diabetes Sci Technol* 2010, 4(4):958-960.
42.Worringham C *et al*: *PLoS One* 2011, 6(2):e14669.
43.Rabin C *et al*: *Telemed J E Health* 2011, 17(10):801-803.
44.Gan KO *et al*: *Aust N Z J Public Health* 2011, 35(3):293-294.
45.Ngo J *et al*: *Br J Nutr* 2009, 101 Suppl 2:S102-112.
46.Wohlers EM *et al*: *Conf Proc IEEE Eng Med Biol Soc* 2009, 2009:5183-5186.
47.Fukuoka Y *et al*: *BMC Public Health* 2011, 11:933.
48.Free C *et al*: *BMC Res Notes* 2010, 3:250.
49.Melanson EL, Jr. *et al*: *Med Sci Sports Exerc* 1995, 27(6):934-940.
50.Freedson PS *et al*: *Med Sci Sports Exerc* 1998, 30(5):777-781.
51.Marshall AL *et al*: *Med Sci Sports Exerc* 2010, 42(6):1094-1102.
52.Martin-Moreno JM *et al*: *Int J Epidemiol* 1993, 22(3):512-519.

Expediente Nº PI13/00618

INVESTIGADOR/A PRINCIPAL: Luis Garcia Ortiz

MEMORIA DE SOLICITUD PROYECTO DE INVESTIGACIÓN EN SALUD
SECCIÓN HIPÓTESIS Y OBJETIVOS

(Ajústese al espacio disponible)

HIPÓTESIS

Hypothesis of the study:

The use of information and communication technologies (ICTs) can help increase physical activity and the number of active people, as well as reduce the number of hours spent sitting. We hope to increase the physical activity of the intervention group by 30 counts/minute or 1 MET/hour per week over the control, and the number of active subjects by 8%, as well as to reduce sitting time by 20 minutes/day.

The use of ICTs can help improve dietary patterns and adherence to the Mediterranean diet. We hope to increase adherence to the Mediterranean diet of the intervention group by 0.5 points over the control group, which would increase by 1.8, and increase the number of individuals in the intervention group over the control that meet at least 9 criteria of the adherence to the Mediterranean diet questionnaire by 8%.

Improvements in eating habits by following a Mediterranean diet, and increasing physical activity to the levels recommended by international institutions can improve cardiovascular risk factors and delay arterial aging.

OBJETIVOS

The overall objective of the first phase of the project is to develop and validate a mobile tool for use in media such as smartphones which people can use quickly and easily to check their lifestyles and receive recommendations on how to make them healthier.

Main objectives

1. To measure the effect of adding an ICT tool, a smartphone application, to a standardized intervention to increase physical activity and to meet the international recommendations of moderate intensity exercise at least 5 days a week for 30 minutes and vigorous intensity for 20 minutes 3 days/week or reach 450 MET • min • week-1. 2. Evaluate the effect of adding an ICT tool, a smartphone application, to a standardized intervention to increase adherence to the nutritional recommendations of the Mediterranean diet and to score at least 9 points on the adherence to the Mediterranean diet questionnaire.

Secondary Objectives

1. To evaluate the effect of the intervention on the improvement of cardiovascular risk factors (clinical blood pressure, weight, lipids, blood glucose and HBA1c, smoking and inflammation markers) and parameters measuring vascular structure and function, (central arterial pressure, augmentation index, pulse wave velocity, cardio-ankle vascular index and carotid intima-media thickness).
2. To evaluate the effect of the intervention on the reduction of sitting time among individuals using the smartphone compared to the control group.
3. To evaluate the effect of the intervention on the reduction of global caloric intake and improved patterns of consumption of macro and micro nutrients.
4. To evaluate the influence of gender on the adherence to the use of the mobile application and on the results of increased exercise or adherence to the Mediterranean diet and arterial aging.

Expediente Nº PI13/00618

INVESTIGADOR/A PRINCIPAL: Luis Garcia Ortiz

MEMORIA DE SOLICITUD PROYECTO DE INVESTIGACIÓN EN SALUD
SECCIÓN PROYECTOS COORDINADOS

En caso de Proyectos Coordinados, el COORDINADOR deberá indicar:
- Objetivos globales del proyecto coordinado, la necesidad de dicha coordinación y el valor añadido que se espera obtener de la misma.
- Objetivos específicos de cada subproyecto (deben están recogidos además en la memoria de cada subproyecto)
- Interacción entre los distintos objetivos, actividades y subproyectos.
- Los mecanismos de coordinación previstos para la eficaz ejecución del proyecto. Máximo 3 páginas.

Expediente Nº PI13/00618
INVESTIGADOR/A PRINCIPAL: Luis Garcia Ortiz

MEMORIA DE SOLICITUD PROYECTO DE INVESTIGACIÓN EN SALUD
SECCIÓN METODOLOGÍA
Diseño, sujetos de estudio, variables, recogida y análisis de datos y limitaciones del estudio. **Máximo 3 páginas.**

DESIGN: This is a randomized, double-blind, multicentre clinical trial aimed at assessing the possible effects of adding an ICT tool (intervention) to support behavioural and educational counselling (control) in increasing physical activity and adapting eating habits to the Mediterranean diet.

SETTING: six REDIAPP groups located in Bilbao, Cuenca, Zaragoza, Valladolid, Barcelona and Salamanca will participate in the study in continuation of the EVIDENT project (PS09 / 00233,01057,01972,01376,0164,01458) implemented in 2010-2012.

STUDY POPULATION: Subjects for the study population will be selected from the EVIDENT project [7], a sample of 1560 subjects randomly obtained from primary care. Those over 70 years of age will be excluded because of their potential difficulties in using ICTs, as will those who cannot exercise or follow a Mediterranean diet. The rest will be randomized centrally from Salamanca using Epidat 4.0 into intervention (IG) and control groups (CG) with a 1/1 ratio. Of the 1560 subjects an estimated 10% will be excluded, leaving a sample of 1350 subjects. An estimated further 10% will decline to participate, so we expect a sample of 1215. Sample size estimation has been performed for the main study variables. Physical exercise, given an alpha risk of 0.05 and a beta risk of 0.10, and SD of 154 would require 1110 subjects (555 per group) to detect an increase of 30 count/min in IG compared to CG. The study will have an alpha risk of 0.05 and a beta of 0.20 to detect an increase of 8% in the number of active subjects in the IG over the CG. Given alpha and beta risks of 0.05 0.10 respectively and a SD of 2, 676 subjects (338 per group) would be required to detect an increase of 0.5 points in the Mediterranean diet (MD) questionnaire in the IG relative to CG. The study will have alpha and beta risks of 0.05 and 0.20 respectively to detect an 8% increase in the number of subjects who meet the MD (9 or more criteria) in the IG compared to the CG. A sample size of 1215 subjects is calculated to have sufficient power to detect clinically relevant differences in the main study variables, given the cluster effect of the design.

VARIABLES AND MEASURING INSTRUMENTS: Most of these were described in Evident I and are published in the protocol [7]. Anthropometric and demographic variables: these are general variables that can modify the intervention effect, such as age, sex, occupation, consumption of tobacco and alcohol, personal history and consumption of drugs; weight, height, waist circumference and clinical blood pressure; blood sampling to measure lipids, blood glucose, renal function and inflammatory markers to assess vascular risk, sample to be frozen for later studies (Salamanca). **Physical activity**. Accelerometer: The main measure of physical activity will be the change in the amount and intensity of physical activity expressed in counts/minute measured by accelerometer. This will also determine the time dedicated to light, moderate and intense activity and kilocalories used. The already validated [49] Actigraph GT3X accelerometers will be employed. The subjects will wear the accelerometer on the right side of the waist, fastened with an elastic belt, for seven consecutive days. The data will be recorded minute by minute. If the accelerometer registers 10 consecutive zeros during 10 minutes, the measurement will be considered null. The intensity of physical activity (light, moderate and intense) will be determined according to Freedson cut-off points [50]. Physical Activity Questionnaire (7-day Physical Activity Recall (PAR)). The 7-day PAR is a general measure of physical activity of proven validity and reliability. It will be administered by trained personnel in a semi-structured interview (10-15 minutes) in which participants provide an estimate of the number of hours spent doing physical or work-related activities that required at least a moderate effort over the previous seven days. The quantity of physical exercise will be estimated in METs/hour/week and people who do at least 30 minutes of moderate activity on 5 days a week, or at least 20 of vigorous activity on 3 days a week, or reach 450 MET·min·wk-1 will be considered active [11] (ACSP and AHA recommendations). **Sitting time questionnaire**: (Marshall) This measures the hours that the individual spends seated during the week while at work, travelling, at home and at the weekend [51]. **Diet. MD adherence questionnaire.** The main outcome variable referring to diet will be measured by the difference in total score (baseline and final) in this questionnaire, validated in Spain and used in the PREDIMED study [26]. It will be administered by trained personnel and uses 14 items to measure compliance with different aspects of DM. Scores ≥ 9 are considered to show good compliance. Assessment of eating patterns: The frequency with which different foods are eaten will be gathered using a questionnaire validated in Spain for of food consumption frequency [52]. It

determines how often foods are eaten and enables estimations of caloric intake and the basic and essential nutrients in the diet to be made.

Mobile tool for the assessment of healthy lifestyles: The instrument being developed is the result of an agreement between the CGB company and the research group GIAPCyL of the REDIAPP (RD12/0005/0004) through the Infosalud Foundation. Software will be developed for smartphone use, with a user-friendly interface which is easy to use for adults, where they can quickly check how their habits measure up to healthy lifestyle recommendations. Food intake will be measured in accordance with standardized models with the aim of establishing both the quantity and the quality of food eaten and its adaptation to the Mediterranean diet, and resulting in the generation of personalized recommendations. Physical activity will be measured by means of an accelerometer included in the application, which will record the steps taken in each 24-hour period, assessing compliance with exercise targets and making recommendations for improvement, as well as allowing physical activity to be included when the phone cannot be used. The extent to which exercise and diet targets have been met will be calculated at the end of the day, and specific recommendations will be made for the following day/days. The information will be stored on the device and downloaded during the control visits. Appendix I contains a report on the technical specifications of the application.

Central blood pressure and peripheral augmentation index (B-pro®) with Pulse Wave Application Software (A-Pulse). **Motivation analysis for change**: The Prochaska and DiClemente model will be used [28] to establish the individual's motivation stage in each interview using the following categories: a) Precontemplation, b) Contemplation, c) Determination, d) Action, e) Maintenance and f) Relapse. **Instrument adherence:** This will be assessed by the Haynes-Sackett test (modified), measuring the number of days and times the device is used. In a sub sample we will also measure: **pulse wave analysis (PWA) and velocity (PWV)** using the SphygmoCor system. **Carotid intima-media thickness (IMT)**: using a SonoSite MicroMaxx ultrasound (Sonocal), and the **cardio-ankle vascular index (CAVI)**: using the Vasera VS-1500® device (Fukuda Denshi).

INTERVENTION: in order to ensure masking, both the common intervention and the specific intervention carried out in the IG will be performed by a researcher other than the one taking the measurements. INTERVENTION COMMON TO BOTH GROUPS: NUTRITION COUNSELLING: Both groups (control and intervention) will receive nutritional advice aimed at good compliance with the MD. The effectiveness of this intervention has been shown in the PREDIMED study [21]. Counselling will consist of an individual 15-minute appointment to explain the ideas behind the MD and emphasize the importance of complying with each of the recommended points. Counselling will be standardized for the two groups and an information leaflet about the session (Appendix III and IV) will be provided. The MD concept will be explained in first part (3 min), while the second part of the session (10 min) will aim to present each particular recommendation with brief and clear messages. Any doubts can be cleared up in the last part of the interview (2 min). PHYSICAL ACTIVITY COUNSELLING: Both groups (control and intervention) will receive advice on physical activity aimed at generating compliance with the current recommendations on physical exercise for the general population. The effectiveness of this intervention has been demonstrated in the PEPAF study [13] and the Osakidetza Prescribe Healthy Life program (PVS). Counselling will consist of an individual 15-minute appointment, where the health benefits of physical activity will be explained and a recommendation of at least 30 min of moderate-intensity activity on 5 days/wk, or 20 min of vigorous-intensity activity on 3 days/wk will be made. Counselling will be standardized for the two groups and an information leaflet about the session (Appendix III and IV) will be provided. In the first part (5 min) the recommendations regarding physical exercise for cardiovascular health will be presented. The second part of the session (8 min) will aim to teach about the intensity of specific types of exercise, such as walking, cycling, and other activities. Any doubts can be cleared up in the last part of the interview (2 min). Counselling aimed specifically at reducing sitting time will be provided.

SPECIFIC STUDY GROUP INTERVENTION: The IG will be given a smartphone for the 3 months duration of the intervention. An initial 15-minute visit will be made in which the subject will be trained in the use of the device, to be used daily for the 3 months. The researcher will train the participant in the use of the tool to measure food intake, to introduce information and to receive recommendations, and in how to use the accelerometer and read the information generated to achieve the recommended 10,000 steps daily. The user must introduce the daily food intake at breakfast, lunch, dinner and in-between snacks, selecting dishes and foods from the application menu. Normal physical activity will be recorded by the device's accelerometer and the user must introduce any activities done without the smartphone (swimming, football, etc). At the end of the day, the application's final daily summary will analyze the intake and exercise balance and generate a recommended plan for the next few days, aimed at improving nutrition and increasing exercise. A further visit will be made a week after device delivery to confirm its

correct use and to clear up any doubts. The smartphone will be collected after three months, at the same time as the review is carried out in both groups.

BASELINE ASSESSMENT AND MONITORING: Baseline assessment: Inclusion criteria will be checked, the project will be explained, the informed consent and the previously reviewed information will be collected and the accelerometer fitted. Visit after 7 days: collection of accelerometer and data of 7-day PAR and Marshall Questionnaires, and food and exercise counselling. Randomization (by a different researcher) will be carried out, and subjects will be informed about the group to which they have been assigned and the follow-up plan. The IG will be given the smartphone and trained in its use. Visit after 15 days: Short visit to the IG to check the device is being used correctly and to clarify doubts. Visit after 3 months: Common assessment of both groups similar to baseline and collection of smartphone. Visit after 12 months: Common assessment of both groups similar to baseline. Masking strategy: The researcher carrying out the randomization and intervention with the IG will be different from the one carrying out the measurements and other interventions (hired staff), who will remain blind throughout the study, as will the researcher performing the data analysis. Given the nature of the study, subjects cannot be blind to the intervention.

STATISTICAL ANALYSIS: The results will be expressed according to the mean ± standard deviation of quantitative, or by frequency distribution in the case of qualitative variables. Results will be subject to an intention-to-treat analysis. The chi-square test will be used to analyze the association between independent qualitative variables, with the McNemar test being used for paired samples. The means between two groups will be compared with the Student's t-test, and the paired Student's t-test will measure change within the same group. The relationship between quantitative variables will be analyzed with the Pearson correlation coefficient. A multivariate analysis of multiple linear regression and logistic regression will be performed to analyze the most determining variables in changes in physical activity and dietary pattern and in arterial stiffness parameters (PWV, AIx and CAVI). Related dependent variables will be analyzed using a GLM. To analyze the effect of the intervention, we will compare the changes registered in the control group with those in the intervention group and calculate the Cohen's d estimate, adjusting for the variables that may influence the result. Logistic regression will be used to analyze the OR of meeting the targets of diet and exercise compliance. A multilevel analysis will be run to determine any effects the different participating centres may have. Gender perspectives will be incorporated in an analysis to assess the differences between men and women in their adherence to the mobile application and the medium and long term results. An analysis will also be made based on the degree of motivation shown in the baseline assessment and on smartphone use adherence. The effect of the intervention may be modified by age, sex, cultural and socioeconomic backgrounds, BMI and some pathologies, as well as baseline lifestyles, which will be controlled for in the analysis. An alpha risk of 0.05 is set for the contrast of hypotheses. The statistical program used will be SPSS v.18.0.

METHODOLOGICAL LIMITATIONS: The study follows all the recommendations of the CONSORT group, but due to the nature of the intervention the participating subjects will not be blind to the intervention. Given that we are dealing with a lifestyle modification, the analysis of the main results of MD is self-reported, although using validated tools and the general food survey can serve as quality control. Nevertheless, objective data (accelerometer) will be available for exercise assessment. Difficulties in using the application may raise the dropout rate in the IG.

ETHICAL ISSUES: The study will be carried out after authorization by the corresponding Ethics Committee, with the prior informed consent of the study subjects and in accordance with the Declaration of Helsinki. The subjects will be informed of the objectives of the project and the potential risks and benefits of the trial to be carried out. None of the research actions involve risks to life for the type of subjects to be included in the study (ethical issues and informed consent are discussed further in Appendix II).

Expediente Nº PI13/00618

INVESTIGADOR/A PRINCIPAL: Luis Garcia Ortiz

MEMORIA DE SOLICITUD PROYECTO DE INVESTIGACIÓN EN SALUD
SECCIÓN PLAN DE TRABAJO

Etapas de desarrollo y distribución de las tareas de todo el equipo investigador, y las asignaciones previstas para el personal técnico que se solicita. Indicar además el lugar/centro de realización del proyecto.

(Ajústese al espacio disponible)

Development stages

The project is planned to run over three years with a subsequent follow-up to evaluate the long-term effects of the intervention. In an early phase in 2013 the software for the smartphone is developed by the computer company CGB and a pre-pilot scheme is run in Salamanca and Barcelona with users of different ages, genres and cultural levels to verify the feasibility of using the tool and to make any necessary changes.

1st quarter of 2014: Equipment purchased. Creation of information collection questionnaire. Preparation of the intervention by the management team. Registration of study protocol in clinical trials and publication of protocol on BMC Public Health. Recruitment of project support staff.

2nd quarter of 2014: Meeting with the PIs for start-up and training of the contracted researchers. Initial visit to all centres and piloting of the project. Piloting is done in all participating centres with 20 subjects from each.

3rd quarter of 2014: Start of the operational phase, with recruitment of subjects and 3-month review. 4th quarter of 2014 and 2015: The inclusion of new patients is completed and the 3-month visit is made. Monitoring visit once all patients have been incorporated. Meeting with PIs and nurses to evaluate follow-up and baseline data analysis and design of baseline data for publication. In the second semester, the final 3-months visits is made and the 12-month visits start. Monitoring visit at the end of the 3-month visit.

2016: Continuation of annual (12-month) visits until completion. Monitoring visit at the end of the 12-month visit. Project closure meeting. Data analysis and plan for the dissemination of final results.

Distribution tasks, Salamanca: The contracted nurse, with the support and supervision of the researchers responsible for this task, will contact the selected individuals and invite them to participate in the study. On a daily basis, she will complete the questionnaires and carry out the research activities relevant to visits 1, 2 and 3 (previously detailed), fitting the accelerometer and processing the information obtained. She will then remove the accelerometer given to the subject the previous week and complete the 7-day PAR and Marshall questionnaires and process the information. She will also be responsible for the management of biological samples and diet and exercise interventions with all subjects. José I Recio Rodríguez (nurse and certified nutritionist), will monitor the study and will be responsible for coordinating the nurses in charge of each group, daily project follow-up in the different participating centres, supervision of the information generated and the quality control of the processes, as well as periodic monitoring, either directly or through external monitoring services. He will also be responsible for the training of contract nurses. The CGB computer company will be in charge of developing the application and analyzing the information generated. Carmela Rodríguez Martín (nurse), will perform randomization and the specific smartphone intervention with those included in the intervention group, as well as the 7-10 day reinforcement visit and the management of biological samples. José A Iglesias Valiente (GP, physical exercise expert) will be in charge of training with regards to assessment and recommendations of physical exercise. Manuel A Gómez Marcos (GP), will participate in the selection and evaluation of participants with special tests, as well as in the dissemination. Mª Carmen Patino Alonso (statistician), has participated in the design of the project and training of participants and will be in charge of analyzing the information and drafting publications. Carmen Castaño Sánchez (psychologist and nurse) and Diana Pérez Arechaederra (psychologist) will contribute expertise regarding the psychological processes to ensure that the way the information is presented has an effect in terms of modifying behaviours. The Department of Physiology at USAL will be in charge of the management and analysis of frozen biological samples. Luis Garcia Ortiz (PI) (GP and postgraduate diploma in statistics) has participated in the design and planning of the study, the training of participants and will supervise the process in all participating centres, ensuring the validity of the information obtained and reviewing the publications generated. He will also participate in the operational phase by carrying out the ultrasound scans.

The project will be implemented in the following health centres: in Salamanca, Valladolid, Bizkaia, Zaragoza, Talavera de la Reina (Toledo) and Barcelona

Expediente Nº PI13/00618

INVESTIGADOR/A PRINCIPAL: Luis Garcia Ortiz

MEMORIA DE SOLICITUD PROYECTO DE INVESTIGACIÓN EN SALUD
SECCIÓN PLAN DE TRABAJO
(Ajústese al espacio disponible. Puede incorporar hasta un máximo de 8 líneas de Actividad/Tarea)

CRONOGRAMA

ACTIVIDAD / TAREA	PERSONA/S INVOLUCRADADAS		E	F	M	A	M	J	J	A	S	O	N	D
		1º Año												
		2º Año												
		3º Año												

ACTIVIDAD / TAREA	PERSONA/S INVOLUCRADADAS		E	F	M	A	M	J	J	A	S	O	N	D
		1º Año												
		2º Año												
		3º Año												

ACTIVIDAD / TAREA	PERSONA/S INVOLUCRADADAS		E	F	M	A	M	J	J	A	S	O	N	D
		1º Año												
		2º Año												
		3º Año												

Expediente Nº PI13/00618

INVESTIGADOR/A PRINCIPAL: Luis Garcia Ortiz

MEMORIA DE SOLICITUD PROYECTO DE INVESTIGACIÓN EN SALUD
SECCIÓN PLAN DE TRABAJO

Inserte (si lo desea) una imagen con un cronograma.

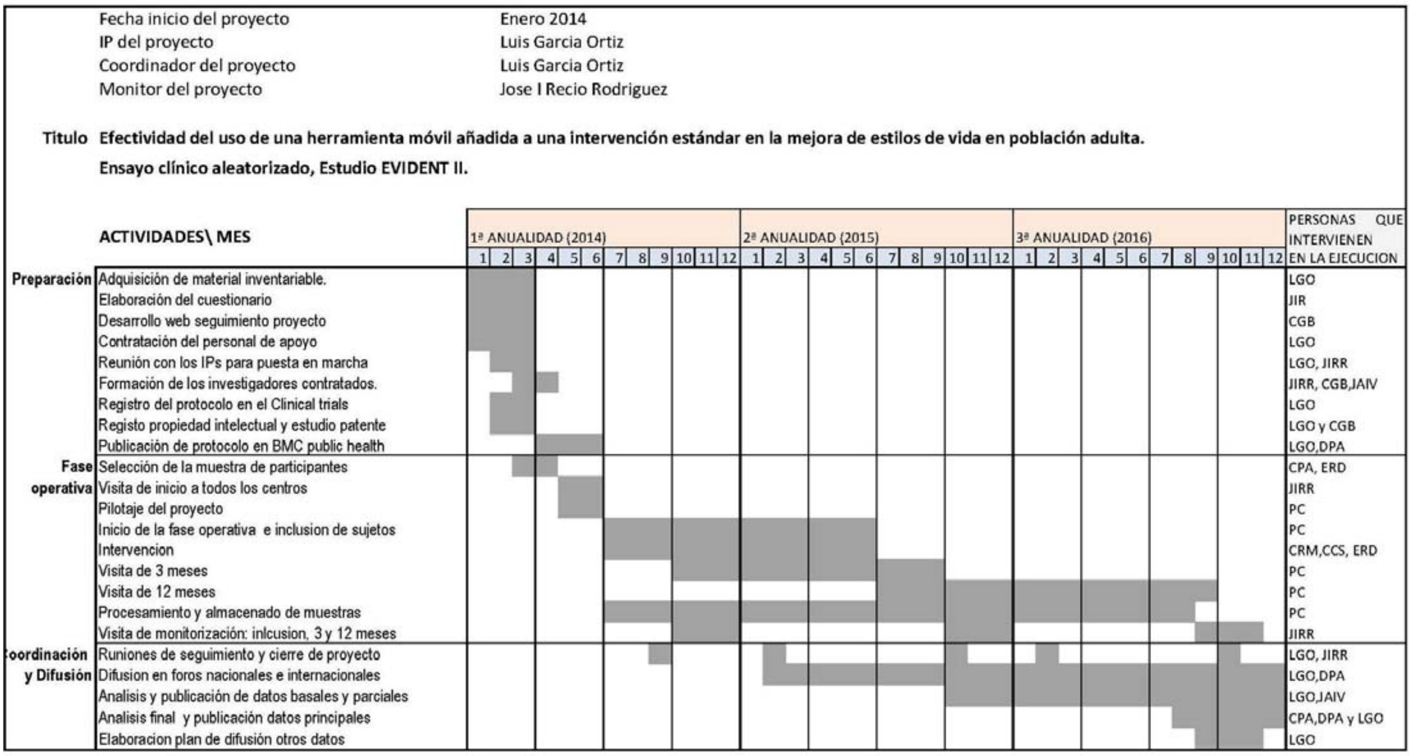

Expediente Nº PI13/00618
INVESTIGADOR/A PRINCIPAL: Luis Garcia Ortiz

MEMORIA DE SOLICITUD PROYECTO DE INVESTIGACIÓN EN SALUD
SECCIÓN EXPERIENCIA DEL EQUIPO

Experiencia del equipo investigador sobre el tema **(Ajústese al espacio disponible)**

PROJECT MANAGEMENT TEAM

The core of the Salamanca team is made up of the lifestyle and cardiovascular risk research group (RD12/0005/0004) which forms part of the Instituto de Salud Carlos III network of preventive activities and promotion of health in primary care (REDIAPP) (RETICS RD12 / 0004) and is integrated in the Instituto Biosanitario de Salamanca (IBSAL). This team has extensive experience in the development of projects, especially in the cardiovascular and lifestyle field. The team participated as a collaborating group on the PEPAF project (PI02/0015) and as a steering group on the EVIDENT project (PS09/00233), which involved 6 network groups and 1500 subjects. This last project has already generated five publications in first and second quartile journals and there are six more under review in journals of the same level, one of them being the validation of one of the measurement instruments, a master's thesis, and 15 national and international papers. This team has experience in the development of computer tools and their application in clinical work (http://www.laalamedilla.org/Investigacion.htm). Together with the CGB company, it has developed several tools for the evaluation of cardiovascular risk and physical activity (three applications for intellectual property rights have been submitted), and with the company Flags Solution it has developed a tool to measure the thickness of retinal vessels (Registration number 00/2011/589) (Journal of Hypertension 2012). Therefore the management team believes that it is sufficiently capacitated for the successful implementation of this project from the scientific and technological points of view. The main lines of research developed in the cardiovascular and lifestyle field are: - Projects of the REDIAPP: PEPAF project (PI02/0015), ISTAPS project (PI021471), ESCAP project (PI051595), EFICAR project (PS09/01337), EVIDENT project (PS09/00233), project MARK (PI10/02043) and design of a complex multi-risk intervention in primary care to encourage health-promoting behaviours (PI12/00616) - CICLO-RISK project: Quality improvement cycles to reduce vascular risk in hypertensive patients. Several projects at autonomous community level. Two doctoral theses and multiple publications and scientific communications have been generated.

LOD-RISK, VASO-RISK and LOD-DIABETES: This line of research studies different methodologies for blood pressure measurement and the usefulness of different instruments for assessing cardiovascular risk through the study of target organ damage (vascular, cardiac and renal) . Three doctoral theses have been read, multiple publications and communications at national and international congresses have been generated. The European 7 PM project is in development. The MID-Frail Study: A randomized clinical trial to evaluate the effectiveness of multi-modal intervention in frail and prefrail older people with type 2 diabetes on frailty and quality of life. In 2012, 24 articles were published, mostly in 1st and 2nd quartile journals and in 2013 saw 8 published and 14 more under review. A line of cooperation with the pharmaceutical industry has been developed through clinical trials and IT companies, and several transfer products have been generated (four intellectual property registrations in 2013). The complete information about the group's research activity can be found online at http://www. laalamedilla.org/Investigacion. htm.

Expediente Nº PI13/00618

INVESTIGADOR/A PRINCIPAL: Luis Garcia Ortiz

MEMORIA DE SOLICITUD PROYECTO DE INVESTIGACIÓN EN SALUD
SECCIÓN MARCO ESTRATÉGICO

(Ajústese al espacio disponible)

1. Capacidad del proyecto de abordar los objetivos, prioridades enmarcadas en el reto Salud, Cambio Demográfico y Bienestar de la Estrategia Española de Ciencia y Tecnología y de Innovación.
2. Capacidad del proyecto de fomentar sinergias e impulsar el talento en el SNS.

1. Capacity of the project to address the objectives and priorities forming part of the Health, Demographic Change and Well-being challenges of the Spanish Strategy for Science, Technology and Innovation. 2. Capacity of the project to foster synergies and boost talent in the national health system.

1.- This project aims in general to address the ultimate goal of R & D & I policies in Spain to improve the health of citizens, in this case through the promotion of healthy lifestyles. In its design, it focuses on the most prevalent health problems, mainly caused by unhealthy lifestyles. The results of the study may therefore contribute to the improvement of the population's health. Mobile phones are used by practically the whole population and the spread of new smartphones as commonly used tools is unstoppable. This project supports the development of technology applied in the field of health; it is aimed not only at developing technology, but above all at demonstrating the utility of this in improving the health of users. Demonstrating that the proper use of this tool can contribute to improving the health of the population, as proposed by the FDA, can give it unlimited potential due to the profusion of smartphones across all sections of the population. Although there are many health-related applications, few have demonstrated that they are able to change lifestyles in the medium and long term, while also improving the health of the population, especially in the prevention of pathologies related to lifestyles, the main chronic diseases affecting western society today. These tools are already influencing new approaches to public health, and health services will have to adapt in the coming years to include them as support in the diagnosis, monitoring and treatment of different health problems. This project involves an important advance in information and communication technologies (ICTs) and contributes to innovation in the area of [illegible]health, and therefore to the development of the national health system. Finally, it will also help assess the impact of new technologies in supporting advice to change to healthier lifestyles. This can contribute to this application and the development of others becoming part of the productive social fabric, and support the transfer of knowledge and wealth in society.

2. Capacity of the project to foster synergies and boost talent in the national health system. This project fosters synergies between the biotechnology and health sectors by promoting joint working groups with a variety of professionals from the computer and health sectors (doctors, nurses, nutritionists, sports doctors, psychologists, etc.). New tools have been developed for the creation of smartphone applications, as well as for measuring aspects of nutrition and physical activity with multi-professional collaboration. Likewise, new collaborative projects have been set up between these sectors that will be developed in the coming years. This is a multi-centre project in which researchers from six health areas and five autonomous communities will participate, with different levels of training and different areas of experience, which brings synergy to the development of the project, especially when it comes to data analysis and dissemination of results. This project will provide selected junior researchers with the opportunity to develop their skills and talents and turn them into future high-impact publications (as was the case with EVIDENT I) and several PhDs. Finally, it provides opportunities to cooperate with technology firms in commercializing the products created, which will be beneficial in terms of obtaining additional funds for the development of new projects. For this purpose, intellectual property rights will be registered and a study of the patentability of the tool developed will be carried out; if it is positive the patents will be processed through the corresponding OTRI (office for transfer of research results) of the Instituto Biosanitario de Salamanca (IBSAL). Finally, an agreement has been reached with the technology firm (CGB Informática) for the development of the application, and a contract/agreement will be drawn up later for its commercialization and generation of funds for future joint projects.

Expediente Nº PI13/00618

INVESTIGADOR/A PRINCIPAL: Luis Garcia Ortiz

MEMORIA DE SOLICITUD PROYECTO DE INVESTIGACIÓN EN SALUD
SECCIÓN MEDIOS DISPONIBLES

(Ajústese al espacio disponible)

Medios disponibles para la realización del proyecto

Resources available for project implementation

At the moment, participating centres have most of the necessary infrastructure for the development of the project at their disposal. Suitable computer equipment and the Teleform system for the preparation of questionnaires and automated reading of data is available at the controlling centre, as is the necessary software for data management and statistical analysis. Technical support is also provided by an epidemiologist and a statistician. The centres are equipped with a physical exercise laboratory and sufficient infrastructure to measure basic anthropometric parameters (Blood pressure, height, weight, waist). For the EVIDENT project, each participating centre was provided with four Actigraph GT3X accelerometers plus Actilife software (Actigraph, Shalimar, FL, USA) to measure physical activity, two Radial Pulse Wave Acquisition Devices (BPro) with Pulse Wave Application Software (A-Pulse) to measure the central and radial pressure augmentation index, and WatchBP Office ABI (Microlife AG [illegible]Swiss Corporation) for automatic calculation of the ankle-arm index. As the basic analytical measurements are routine parameters, they will be determined in the corresponding reference centres. The analysis of biological markers will be carried out with the Salamanca patient sample. Centrifuge and freezer are available there for the handling of biological samples and their subsequent analysis in the department of physiology and medicine of the University of Salamanca, where they will be sent by a courier service with project-independent financing. The Salamanca unit is equipped with the SphygmoCor System for the analysis of pulse wave velocity and central augmentation index in the subsample of this group. An ultrasound unit is available for measuring the average carotid intima-media thickness in Salamanca and Cuenca. Salamanca and Barcelona have the VASERA device for the calculation of the Cardio Ankle Vascular Index.

Therefore, it would be necessary to acquire only: 25 smartphones in each group and 4 more accelerometers to keep the operation effective, plus the software update for BPro and APulse.

Smartphone application: The development of the application to the specific requirements of the research project will be financed through an agreement of the Infosalud Foundation and CGB Computers.

Expediente Nº PI13/00618

INVESTIGADOR/A PRINCIPAL: Luis Garcia Ortiz

MEMORIA DE SOLICITUD PROYECTO DE INVESTIGACIÓN EN SALUD
SECCIÓN JUSTIFICACIÓN DETALLADA DE LAS PARTIDAS PRESUPUESTARIOS SOLICITADAS
(Ajústese al espacio disponible)

1. Staff Costs

2. Operational Costs

a) Acquisition of goods and contracting of services: €48,060

- Nursing technical support services: €36,750 To ensure the effective implementation of the project, it is necessary to hire certain support services for research personnel, such as data collection, patient schedule management and certain operational actions in which the participation of the research staff is not essential given that most of the researchers are full-time care professionals. In addition to carrying out the anamnesis of habits and case history, these actions include the measurements of weight, height, waist circumference, clinical blood pressure, central arterial pressure, pulse wave velocity, radial augmentation index, ankle/arm index, the fitting and removal of accelerometers, the exercise and the nutritional pattern assessment (questionnaires on activity, sedentary lifestyle and adherence to the Mediterranean diet and dietary pattern) and lifestyle intervention (diet and exercise). Taking into account that approximately 115-125 patients in the control group and a further 115-125 in the intervention group are involved at each node, the four visits (plus extra visits for accelerometer collection and questionnaires) involve an estimated average time per patient of 8 hours, covering information collection, explorations and data recording, which in turn means that the project runs to a total of 1900 hours. To this must be added telephone patient scheduling, the computerization of data using Teleform, as well as training and monitoring activities. The estimated average time for the implementation of the program would therefore be between 2100 and 2300 hours, which is equivalent to about half a day every workday for three years. The suitable professional would have a diploma in nursing and the estimated approximate time involved is equivalent to 6 months of full time work per year over the three years of the project (equal to €12,250/year, in total €36,750).

-Equipment costs: €6,450. Most of the infrastructure necessary for the implementation of the project is currently already available, but four more accelerometers will be needed to supplement the four available to avoid slowing down the rate of data capture and measurement: • 4 Actigraph GT3X Accelerometers (Actigraph, Shalimar, FL, USA), at €425 each = €1,700. • 25 HTC Desire smartphones with SD card and Android operating system at €190 each = €4,750

- Cost of consumables: €2,360 • To measure food intake frequency, University of Navarra questionnaires (Predimed) will be used, which were already used in EVIDENT I. Each questionnaire including analysis costs €3, so for 480 (240 baseline and 240 final) the total cost for each group will be €1,440. • The BPro and A-Pulse software, purchased for the EVIDENT I project, requires updating: €920

- Coordination services (for the coordinating group only): €2,500. For the proper running of the project, a series of coordination and monitoring services is required, such as the development of a website for project monitoring, management of information generated by the smartphone in the different groups and the specific costs involved in the data monitoring process. The estimated total budget for this is €2,500.

b) Travel expenses: €700. An initial meeting will be held in Madrid with the PI, the researcher carrying out the intervention and the researcher hired for the project to explain the methodology and implementation of the project. The estimated cost of travel and hotel would be €100 per person, total €300. The coordination meetings will be attended by at least the PI and the contracted nurse. There will be a final meeting in Madrid to close the project and draw up the publication plan, costing an estimated €200 in the second year and a further €200 in the third year.

Expediente Nº
PI13/00618

INVESTIGADOR/A PRINCIPAL: Luis Garcia Ortiz

MEMORIA DE SOLICITUD PROYECTO DE INVESTIGACIÓN EN SALUD
SECCIÓN PRESUPUESTO

Presupuesto solicitado

1. Gastos de Personal **Euros**

Subtotal Gastos de Personal :

2. Gastos de Ejecución

A) Adquisición de bienes y contratación de servicios

(Bienes inventariable, material fungible y otros gastos)

Nursing technical support services	36.750
Equipment costs	6.450
Cost of consumables	2.360
Coordination services	2.500
**Subtotal Gastos Bienes y Servicios :**	**48.060**

B) Gastos de Viajes

Travel expenses	700
**Subtotal Gastos Viajes :**	**700**
**Subtotal Gastos Ejecución :**	**48.760**
**Total Presupuesto :**	**48.760**

Expediente Nº PI13/00618

INVESTIGADOR/A PRINCIPAL: Luis Garcia Ortiz

MEMORIA DE SOLICITUD PROYECTO DE INVESTIGACIÓN EN SALUD
SECCIÓN ANEXOS

INTRODUZCA TEXTO COMO ANEXO **Máximo 3 páginas.**

APPENDIX I: TECHNICAL REPORT ON THE SMARTPHONE APPLICATION

Software Product Lines (SPL) combine systematic development with reuse of coarse-grained components that include the common and variable parts of the product. We propose that a standard UML package mix is used to represent an SPL architecture with conventional tools and variations, as well as some of the most widespread and consolidated design patterns within the software development industry such as MVC, BO, DAO and VO, thereby achieving great advantages such as reuse of components, easy structuring of the application, greater scalability, ease of testing, and easier future maintenance of the application. The product is initially intended as a development for Android devices, so Java, Eclipse and Android SDK platforms will be used, applying the principles mentioned above at all times. There will be a differentiation between the presentation layer (where XML files and different resources will define the views offered by our application) and the core, where its functionality will lie. This functionality will be totally implemented in Java and grouped in packets. In each packet we will group all those classes placed under the same module or functional framework, thus bringing together all the elements of our code that model a well differentiated common characteristic. Looking towards the possible international rollout of the application and thus its easy translation in the future, both the text and image resources will be separated in folders that follow the same nomenclature, assigning each of them their language through the corresponding abbreviation in accordance with ISO 693-1. Thus, depending on the language in which the terminal is configured, the application will automatically draw from the elements defined for that language, without having to configure it explicitly. As for the application modules, the following are set: Connection and access to the database management system: the application data is stored in a local SQLite database, especially suited to the technology employed since it is a small scale system that does not require configuration and is also available on all devices with an Android operating system. DAO and VO design patterns will be used, thanks to which we will encapsulate access to the database for later use in the business logic layer. Pedometer: The calculation of physical exercise through the pedometer will be located in a service, a component used for long-running operations running in the background without a user interface, making it independent of the rest of the application (where the user does interact with it). This service will collect data from the accelerometer, a hardware sensor built into the vast majority of devices with Android technology which detects acceleration. To transform the values ��returned by this sensor into steps performed by the user, an algorithm will be implemented based on a study of the characteristics of the steps that a person takes in relation to the axial values ��"x, z, y" provided by the sensor, alongside the configurable values ��regarding the sensitivity that we want the sensor to have. Being an integrated component of the application but with an independent identity, the communication between the service and the main activity of the application will be managed using handler instances, with which we will notify the main activity of the result of the service's calculations (detected steps). Notifications: sampled data originating both from manual entries by the user and from the service, where the pedometer is constantly operating, is stored in the database, thanks to which customized calculations can be carried out, depending on physical characteristics. Based on these calculations, different programmed messages will be displayed either daily or weekly, informing the user of their food and exercise patterns, alongside various recommendations. In order to allow timely planning of these messages, we will use broadcast receivers, which are Android components set up to detect certain events (in our case, a certain day and time), so the application will be launched according to schedule and displaying the appropriate notification. A main module housing the different activities of the application will act as an interface between the different parts. Within this module, we can group functionality into four parts: -Diet: where the data regarding the user's daily intake are collected. -Physical activity: here the user will introduce the different activities that s/he performs throughout the day, unless already included in the calculations made by the pedometer. -Calendar: this serves as a link between the various options of the application, but differentiating them over time. -Configuration-Administration: where user data and different parameters are defined that will then influence the calculations and recommendation messages. Additionally, by running a unit test project, we will be able to verify the correct functioning of the different components of the application, thus guaranteeing its robustness and reliability. To this end, we will use Robolectric together with Maven (POM-based software tool for project management and construction).

APPENDIX II: ETHICAL ISSUES AND GENERAL INFORMED CONSENT

The study will be implemented after the authorization of the corresponding Ethics Committee, once informed consent of the study subjects is given, and in accordance with the Declaration of Helsinki. The subjects will be informed of the project goals and the risks and benefits of the actions to be carried out. None of the actions involve risks to life for the type of subjects to be included in the study (see informed consent in Appendix III). As part of the study, biological samples are obtained and stored (only in Salamanca), so the study subjects will be thoroughly informed. The samples will continue to be traceable until the longitudinal study is completed, and then irreversibly dissociated from personal data or destroyed, in accordance with the

wishes of the subject. This study will serve as a basis for future studies within the framework of the overall objectives, both of our group and of other groups that access the sample biobank of the Instituto Biosanitario de Salamanca (IBSAL). In the light of the above, the confidentiality of the subjects included in accordance with the Organic Law for the Protection of Personal Data (15/1999, December 13, LOPD) will be guaranteed at all times, and under the conditions established by Law 14/2007 of biomedical research.

APPENDIX III: GENERAL INFORMED CONSENT

Effectiveness of a smartphone application for improving healthy lifestyles. (EVIDENT II) You are invited to take part in the current study, which is the next phase of the EVIDENT study in which you have recently participated. Before confirming your participation in this research study, it is important that you understand what it involves. Please read this document carefully and ask any questions you may have. Objective of the study: To evaluate the effect of adding a mobile phone application to the usual advice in order to increase adherence to the nutritional recommendations of the Mediterranean diet and increase physical activity levels to meet international recommendations. At the same time, the effects of lifestyles changes on arterial aging will be studied. Study procedures: The physician/researcher will assess whether you are a suitable candidate for this study. Once you have given your consent and the researcher has checked that you meet the criteria for participation in the study, you will be asked some questions about your health, physical activity and nutrition, and the following tests will be performed: Measurements of weight, height, waist circumference, central and peripheral blood pressure and ankle/arm index. Physical activity levels are measured with a survey and an accelerometer, a device that evaluates ordinary activity performed during a week. Your nutritional pattern is assessed through a self-reported dietary survey. A blood sample will be taken to determine blood count and biochemistry. Participants will be randomly distributed in two groups. One of these is the control group, which will receive counselling on nutrition and exercise that has been proven effective in previous studies, and the other is the intervention group, which besides being provided with the above will also be given a mobile phone with an application for nutrition and exercise which is to be used daily for 3 months. Visits will be made after one week, three months and twelve months in order to check compliance with recommendations regarding diet and physical exercise, evaluate risk factors and measure arterial aging. Expected benefits and potential risks: In addition to knowing your peripheral and central arterial pressure, you will benefit from learning about other cardiovascular risk factors and the state of arterial aging, and you will receive proven standardized advice for improving your lifestyles (exercise and food). If you are in the intervention group you will receive a mobile phone that will make meeting the recommendations easier. You will also receive a detailed report with the results of the investigations performed. These research actions do not involve any risk to life, only the discomfort that may result from doing the tests themselves (half an hour), none of which are invasive, and wearing the accelerometer for a week. Confidentiality: If you agree to take part in this study, please note that some information about your health will be used and will be incorporated into a computerized database without your name. No patient will be personally identified in the dissemination and publication of the results. Your medical records may be checked by employees of the health authorities, independent ethics committees and other persons designated by law to verify that the study is being performed correctly. All your data will be kept under strict confidentiality and cannot be divulged by any means; doctor-patient confidentiality is maintained at all times (Data Protection Act 15/1999). Any unforeseen problems or medical emergencies, either based on existing issues or arising during the course of the study, will be dealt with. The study will be interrupted if other therapeutic priorities arise. Commitment of participation: I .... (name and surname of the participant) have read the information sheet given to me. I have received enough information and have had the opportunity to ask questions about the study. I have spoken with .... (name and surname of the health professional). I understand that my participation is voluntary and that I can withdraw from the study at any time, without having to give explanations and without this affecting my medical care. I hereby freely agree to participate in the study. Signed, in ...., on the .... of .... of 20 ... Name and surname of the participant Signature Date. Specific authorization for the freezing of serum. (Salamanca only). Signed: (participant) Mr/Ms ...., doctor/investigator, I have informed the signatory of all the above, clarifying his/her doubts and acknowledging his/her understanding of all the information presented. Name and surnames of the health professional. Signature Date

SECTION FOR REVOCATION OF CONSENT. I, ..., revoke my consent to participate in the study indicated above. Signature: Date: .... / .... / .... Questions/Information If you have any questions or would like to clarify any issues related to the study, or if you need help with any health issues related to this study, please do not hesitate to contact:

ANEXO III.1: MEDITERRANEAN DIET ADVICE

1-Use olive oil as the main fat for cooking. Its content in vitamin E, beta-carotenes and monounsaturated fatty acids confer cardioprotective properties. It also gives the dishes a unique flavor and aroma. 2-Consume foods of vegetable origin in abundance: fruits, vegetables, legumes and nuts. Vegetables, vegetables and fruits are the main source of vitamins, minerals and fiber in our diet. It is essential to consume 5 servings of fruit and vegetables daily. Its content in antioxidants and fiber can contribute to prevent some cardiovascular diseases. Fresh and seasonal foods are the most suitable because it allows us to consume them at their best, both in terms of nutrients and their aroma and flavor. 3-Bread and food from cereals (pasta, rice and especially their whole products) Your daily consumption is indispensable because of its rich composition of carbohydrates. They give us an important part of the energy needed for our daily activities. Its integral products give us more fiber, minerals

and vitamins. 4- Consume daily dairy products, mainly yogurt and cheeses. For being excellent sources of proteins of high biological value, minerals (calcium, phosphorus, etc.) and vitamins. 5-Consume preferably white meat (chicken, turkey, rabbit) than red meat (veal, sausages) Recommend consumption in small quantities, preferably lean meats, and forming part of dishes based on vegetables and cereals. 6-Eat plenty of fish and eggs in moderation. Consumption of blue fish at least 1-2 times a week for having properties very similar to fats of plant origin to which they are attributed protective properties against cardiovascular diseases. Consumption of three or four eggs a week is a good alternative to meat and fish. 7- Sweets and cakes should be consumed occasionally. 8-Wine should be taken in moderation and during meals. It can have beneficial health effects by consuming it in moderation and in the context of a balanced diet.

INTRODUZCA IMÁGENES COMO ANEXO **Máximo 1 página.**

evident

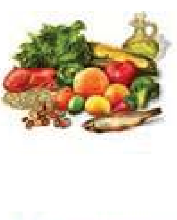

Es bien sabido que en los países ribereños del mar Mediterráneo ha habido tradicionalmente una baja frecuencia de enfermedades del corazón y de muertes por esta causa, junto con una larga esperanza de vida. Se cree que esto es debido, entre otros factores, a los hábitos alimentarios tradicionales de las gentes que viven en la zona del Mediterráneo.

La dieta mediterránea se caracteriza por tener como base de la alimentación el aceite de oliva y los cereales (trigo), aunque a nivel nutricional destacan mas alimentos: fruta fresca, verduras, hortalizas, frutos secos, legumbres, una moderada cantidad de vino tinto y una alimentación en la que predomine el pescado sobre la carne.

Recomendaciones para seguir una dieta mediterránea.

- Utilice aceite de oliva para cocinar y aliñar los alimentos
- Consuma 2 o mas raciones al dia de verduras y hortalizas (1 como minimo cruda en ensalada)
- Tome 3 o mas piezas de fruta al dia (incluyendo zumos naturales)
- Consuma legumbres al menos 3 veces por semana
- Consuma pescados o mariscos al menos 3 veces a la semana (1 como minimo de pescado azul)
- Tome frutos secos al menos 3 veces por semana
- Consuma con mayor frecuencia carnes blancas (aves sin piel o conejo) que carnes rojas, embutidos u otras carnes procesadas.
- Aderece los platos de pasta, arroz, y verduras con sofritos de tomate, ajo, cebolla o puerro al menos 2 veces ala semana.
- Limite a menos de una vez a la semana el consumo de los siguientes alimentos y bebidas:
  - -Nata, mantequilla o margarina.
  - -Bebidas azucaradas (refrescos)
  - -Reposteria o bolleria industrial, patatas de bolsa y alimentos o platos precocinados.

evident

Los beneficios de la actividad física, tanto a nivel biológico, como psicológicos, van desde una mejor calidad de vida, a la reducción de los riesgos asociados a la hipertensión, obesidad y diabetes, o a la prevención y control de enfermedades cardiovasculares, osteoporosis, ansiedad, depresión y algunos cánceres.

El tiempo necesario depende de la intensidad del esfuerzo

	Ejercicio Moderado (30 – 60 minutos)	Ejercicio Vigoroso (20 – 30 minutos)
**Actividad**	Caminar a paso rápido<br>Natación<br>Bicicleta<br>Bailar	Footing<br>Aerobic<br>Fútbol, Baloncesto, Tenis...
**Frecuencia**	5 a 7 veces/semana	3 o más veces/semana

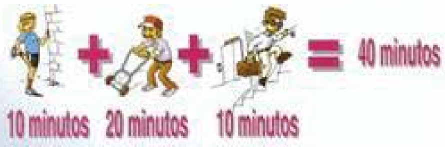

Consejos sobre actividad fisica

Tabla 16.3.- Dosis minima de los elementos de la prescripcion de ejercicio para inicio y mantenimiento

	Tipo de actividad física (ver tabla de actividades)			
	Aeróbica: moderada, vigorosa o una combinación de ambas		Ejercicios de fuerza	Ejercicios de flexibilidad
Intensidad	Moderada<br>FCE > 60% FC máxima (45% FC de reserva)	Vigorosa<br>FCE > 80% FC máxima (60% FC de reserva)	Cargas (peso) que produzcan fatiga al cabo de 8-12 repeticiones	Movilidad articular: hasta los topes<br>Estiramientos: sensación de tirantez soportable
Duración mínima ***recomendada***	Acumular 30 minutos/día<br>≥ 3 periodos de 10 minutos<br>150 minutos/semana	Acumular 20 minutos/día<br>≥ 2 periodos de 10 minutos<br>75 minutos/semana	8 repeticiones de 8 ejercicios	Movilidad articular: 10 repeticiones<br>Estiramientos: mantener la tirantez 10 segundos
Frecuencia mínima	5 días/semana	3 días/semana	2 días/semana no consecutivos	2 días/semana no consecutivos
Dosis ***inicial*** en personas totalmente inactivas, progresión y ***mantenimiento***	La dosis de inicio del plan puede ser inferior a la mínima recomendada, ej.: 10 minutos/sesión x 2 días/semana<br>Incremento quincenal de 5 minutos/sesión o mensual de 1 día/semana hasta dosis mínima en 3 meses<br>Progresión a 6 meses: incrementar quincenalmente la actividad aeróbica hasta doblar la dosis mínima			

Tomado del manual de intervención Prescribe vida saludable (Osakidetza), basado en el estudio PEPAF.

evident

Efectividad del uso de una aplicación para Smartphone en la mejora de estilos de vida saludables. Ensayo clínico aleatorio (EVIDENT II).
