# Supplementary figures and images for: Long-Term Effectiveness of a Smartphone App for Improving Healthy Lifestyles in General Population in Primary Care: Randomized Controlled Trial (Evident II Study)
Source: JMIR Mhealth Uhealth. 2018 Apr 27;6(4):e107. doi: 10.2196/mhealth.9218 (PMC5948409; doi:10.2196/mhealth.9218)

Main screen and physical activity screen

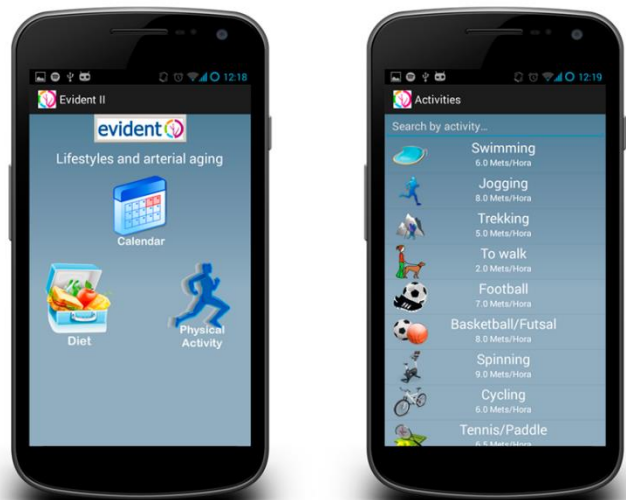

Nutrition screen

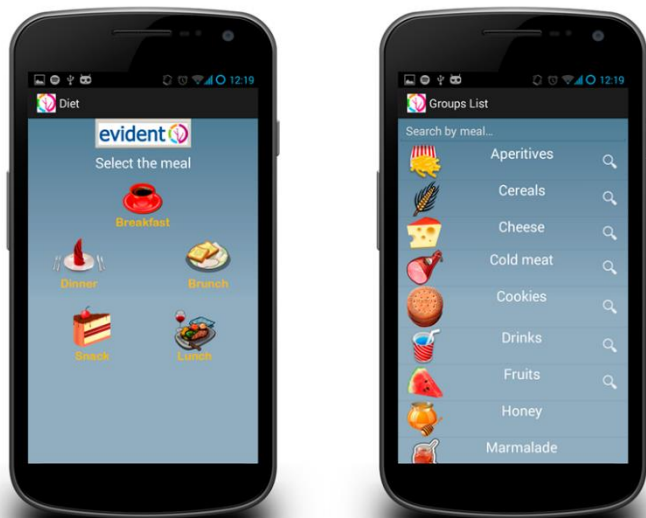

Feedback screen

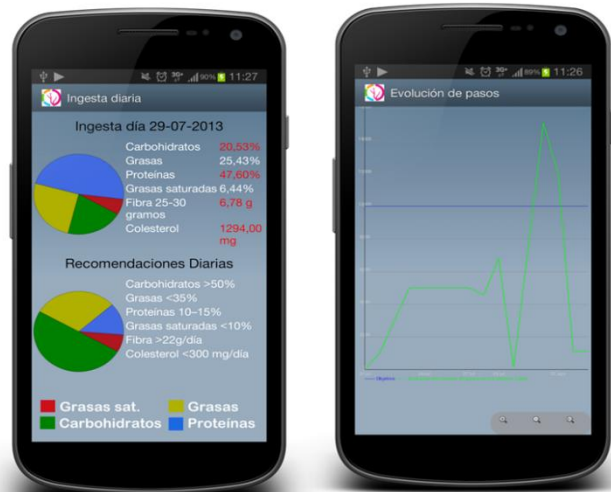

Supplement: Multimedia Appendix 2 [file mhealth_v6i4e107_app2.pdf]
