# Supplementary material for: Long-Term Effectiveness of a Smartphone App for Improving Healthy Lifestyles in General Population in Primary Care: Randomized Controlled Trial (Evident II Study)
Source: JMIR Mhealth Uhealth. 2018 Apr 27;6(4):e107. doi: 10.2196/mhealth.9218 (PMC5948409; doi:10.2196/mhealth.9218)
Supplement: Multimedia Appendix 3 [file mhealth_v6i4e107_app3.pdf]

	Baseline		Changes within groups from baseline to 12 months			Comparing changes from baseline between intervention and control groups		
**Accelerometer**	Group	Mean	SD	Mean difference	95% CI	*P* adjusted	Mean difference	95% CI	*P* adjusted
Steps per day	1	9992	3847	−1625	−2033, −1217	<.001	-519.9	−1018.8, −21.0	.04
	2	9708	3931	−956	−1357, −554	<.001			
Counts minutes per week	1	69	70	−21	−29, −13	<.001	−8.5	−16.8, −0.2	.04
	2	66	69	−10	−17, −3	.007			
Sedentary minutes per week	1	8327	540	180	118, 242	<.001	53.7	−27.0, 134.4	.19
	2	8341	526	116	58, 174	<.001			
Light minutes per week	1	1298	437	−88	−141, −35	.001	−19.4	−89.3, 50.5	.58
	2	1307	423	−65	−115, −15	.011			
Moderate minutes per week	1	438	205	−85	−108, −62	<.001	−25.4	−52.7, 1.9	.07
	2	413	213	−48	−70, −26	<.001			
Minutes of vigorous or very vigorous activity per week	1	17	39	−7	−11, −3	<.001	−4.9	−9.7, 0.0	.04
	2	18	46	−2	−7, 2	.31			
Total MVPA minutes per week	1	455	216	−93	−118, −68	<.001	−30.2	−58.7, −1.7	.04
	2	433	222	−51	−74, −29	<.001			

MET minutes per week	1	1851	892	−375	−476, −274	<.001	−128.3	−246.4, −10.2	.03
	2	1763	922	−201	−296, −107	<.001			
**7-day PAR questionnaire**									
Minutes of moderate activity per week	1	153	265	2	−26, 29	.89	0.2	−30.6, 31.0	.99
	2	155	258	−1	−28, 26	.96			
Minutes of moderate activity in leisure time per week	1	131	213	14	−10, 39	.24	2.9	−26.3, 32.0	.85
	2	148	249	2	−22, 27	.85			
Minutes of vigorous or very vigorous activity per week	1	30	99	−13	−23, −3	.01	−8.3	−18.8, 2.3	.12
	2	30	106	−7	−19, 6	.29			
Minutes of vigorous or very vigorous activity in leisure time per week	1	28	97	−12	−22, −2	.01	−9.2	−19.7, 1.4	.08
	2	28	98	−4	−17, 8	.47			
Minutes of MVPA per week	1	183	293	−11	−41, 18	.44	−7.7	−39.2, 23.8	.63
	2	185	285	−7	−36, 21	.61			

		Minutes of MVPA in leisure time per week	1	159	229	2	−23, 27	.88	−5.8	−35.5, 23.9	.70
			2	176	272	−2	−28, 24	.88			
		MET minutes per week	1	865	1408	−116	−258, 25	.11	−59.9	−199.8, 80.0	.40
			2	866	1331	−70	−206, 66	.31			
		MET minutes per week in leisure time	1	764	1120	−58	−177, 60	.33	−56.8	−189.8, 76.2	.40
			2	826	1263	−44	−169, 80	.48			
