# Supplementary material for: Long-Term Effectiveness of a Smartphone App for Improving Healthy Lifestyles in General Population in Primary Care: Randomized Controlled Trial (Evident II Study)
Source: JMIR Mhealth Uhealth. 2018 Apr 27;6(4):e107. doi: 10.2196/mhealth.9218 (PMC5948409; doi:10.2196/mhealth.9218)
Supplement: Multimedia Appendix 4 [file mhealth_v6i4e107_app4.pdf]

Criteria				Baseline		Changes within groups from baseline to 12 months			Comparing changes from baseline between intervention and control groups		
	**Mediterranean diet**		Group	Mean (N)	SD (%)	Mean difference	95% CI	*P* adjusted	Mean difference	95% CI	*P* adjusted
		Using olive oil as the principal source of fat for cooking	1	389	94	2.3	0.0, 4.5	.04	−0.1	−2.5, 2.2	.90
			2	394	94	2.5	0.3, 4.7	.03			
		Having ≥4 tbsp (54 g) of olive oil per day (eg, used for frying, in salads, meals eaten from home)	1	154	37	4.0	−1.5, 9.5	.15	3.3	−3.2, 9.8	.32
			2	135	32	3.9	−1.4, 9.1	.15			
		Having 2 or more servings of vegetables per day	1	166	40	10.0	4.3, 15.7	.001	1.6	−5.1, 8.3	.63
			2	151	36	11.6	6.2, 17.0	<.001			
		Having 3 or more pieces of fruit per day	1	180	43	4.8	−0.4, 10.1	.07	−0.4	−6.5, 5.8	.90
			2	177	42	6.9	2.3, 11.5	.003			
		Having 1 serving of red meat or sausage per day	1	347	84	3.7	−0.5, 7.9	.08	−1.7	−5.8, 2.4	.42
			2	355	85	5.2	2.0, 8.5	.002			
		Having 1 serving of animal fat per day	1	378	91	2.6	−0.5, 5.7	.11	−1.7	−4.7, 1.4	.28
			2	379	91	4.4	1.7, 7.1	.002			
		Having 1 cup (100 mL) of sugar-sweetened beverages per day	1	356	86	3.4	−0.2, 7.0	.06	−1.4	−5.2, 2.5	.48
			2	363	87	3.0	−0.2, 6.2	.06			
		Having ≥7 servings of red wine per week	1	79	19	−1.4	−4.7, 1.9	.39	−1.4	−5.2, 2.5	.49
			2	70	17	0.3	−2.3, 2.9	.83			

		Having ≥3 servings of legumes per week	1	98	24	−2.3	−6.6, 2.1	.30	−0.9	−6.0, 4.2	.73
			2	81	19	0.0	−4.1, 4.1	1.0			
		Having ≥3 servings of fish per week	1	163	39	3.4	−1.9, 8.7	.20	2.0	−4.3, 8.3	.53
			2	183	44	−0.3	−5.2, 4.6	.91			
		Having <2 commercial pastries per week	1	206	50	6.8	1.6, 12.0	.01	0.2	−6.4, 6.7	.96
			2	195	47	8.9	3.2, 14.6	.002			
		Having ≥3 servings of nuts per week	1	149	36	−2.0	−7.2, 3.2	.45	−9.0	−15.3, −2.7	.005
			2	123	29	10.2	5.1, 15.3	<.001			
		Preferring white meat over red meat	1	282	68	7.7	2.8, 12.6	.002	−4.4	−9.9, 1.2	.12
			2	261	62	14.7	10.0, 19.4	<.001			
		Having ≥2 servings per week of a dish with a traditional sauce of tomatoes, garlic, onion, or leeks sautéed in olive oil	1	220	53	2.3	−3.3, 7.8	.42	2.7	−4.0, 9.4	.43
			2	223	53	−0.3	−6.1, 5.5	.93			
		Score for adherence to Mediterranean diet (mean±SD)	1	7.6	2.1	0.4	0.2, 0.6	<.001	−0.13	−0.37, 0.10	.26
			2	7.4	2.0	0.7	0.5, 0.9	<.001			
		Study participants with a total score ≥9 points (n%)	1	142	34	9.1	3.6, 14.6	.001	0.8	−5.8, 7.4	.82
			2	119	28	12.1	6.8, 17.4	<.01			
