# Supplementary material for: Long-Term Effectiveness of a Smartphone App for Improving Healthy Lifestyles in General Population in Primary Care: Randomized Controlled Trial (Evident II Study)
Source: JMIR Mhealth Uhealth. 2018 Apr 27;6(4):e107. doi: 10.2196/mhealth.9218 (PMC5948409; doi:10.2196/mhealth.9218)
Supplement: Multimedia Appendix 5 [file mhealth_v6i4e107_app5.pdf]

			Baseline		Changes within groups from baseline to 12 months			Comparing changes from baseline between intervention and control groups		
**Accelerometer**		Group	Mean	SD	Mean difference	95% CI	*P* adjusted	Mean difference	95% CI	*P* adjusted
	Steps per day	1	10638	3767	−1055	−1502, −609	<.001	1500 .1	773 .2, 2227 .0	<.001
		2	9513	3818	−2555	−3126, −1984	<.001			
	Counts minutes per week	1	78	70	−13	−20, −6	.002	21 .3	9 .2, 33 .3	.001
		2	61	71	−34	−44, −25	<.001			
	Sedentary minutes per week	1	8284	495	131	55, 206	<.001	−130 .4	−253 .0, −7 .9	.04
		2	8362	568	261	165, 357	<.001			
	Light minutes per week	1	1310	407	−66	−131, 0	.040	58 .2	−48 .1, 164 .4	.28
		2	1286	453	−124	−208, −40	.01			
	Moderate minutes per week	1	469	204	−54	−79, −29	<.001	81 .7	41 .1, 122 .2	<.001
		2	415	199	−136	−168, −104	<.001			
	Minutes of vigorous or very vigorous activity per week	1	17	40	−7	−10, −3	.02	1 .3	−4 .5, 7 .2	.66
		2	17	41	−8	−13, −3	.007			
	Total MVPA minutes per week	1	487	212	−61	−87, −35	<.001	83 .2	40 .7, 125 .6	<.001
		2	433	215	−145	−178, −111	<.001			
	MET minutes per week	1	1981	875	−244	−351, −136	<.001	343 .6	168 .8, 518 .3	<.001

			2	1763	899	−587	−724, −450	<.001			
	**7-day PAR questionnaire**										
		Minutes of moderate activity per week	1	174	290	9	−18, 36	.77	17 .5	−26 .2, 61 .3	.43
			2	124	224	−9	−43, 25	.38			
		Minutes of moderate activity in leisure time per week	1	153	228	18	−8, 44	.71	9 .2	−33 .7, 52 .1	.67
			2	103	188	9	−25, 42	.17			
		Minutes of vigorous or very vigorous activity per week	1	29	103	−16	−23, −9	.04	−7 .2	−18 .2, 3 .7	.19
			2	30	94	−9	−17, 0	.12			
		Minutes of vigorous or very vigorous activity in leisure time per week	1	28	103	−15	−22, −9	.048	−7 .6	−18 .3, 3 .2	.17
			2	27	90	−8	−16, 1	.15			
		Minutes of MVPA per week	1	204	324	−6	−33, 21	.37	13 .8	−29 .7, 57 .4	.53
			2	154	244	−20	−54, 14	.93			
		Minutes of MVPA in leisure time per week	1	181	245	3	−23, 30	.64	3 .9	−38 .7, 46 .5	.86
			2	130	202	−1	−34, 33	.41			
		MET minutes per week	1	946	1573	−98	−214, 19	.15	49 .3	−139 .5, 238 .1	.61
			2	757	1149	−147	−295, 1	.46			
		MET minutes per week in leisure time	1	849	1198	−54	−168, 60	.21	12 .5	−171 .9, 196 .8	.89
			2	653	1000	−66	−211, 79	.97			
