# Supplementary material for: Long-Term Effectiveness of a Smartphone App for Improving Healthy Lifestyles in General Population in Primary Care: Randomized Controlled Trial (Evident II Study)
Source: JMIR Mhealth Uhealth. 2018 Apr 27;6(4):e107. doi: 10.2196/mhealth.9218 (PMC5948409; doi:10.2196/mhealth.9218)
Supplement: Multimedia Appendix 6 [file mhealth_v6i4e107_app6.pdf]

Criteria	Group	Baseline Mean (N)	Baseline SD (%)	Changes within groups from baseline to 12 months: Mean difference	95% CI	*P* adjusted	Comparing changes from baseline between intervention and control groups: Mean difference	95% CI	*P* adjusted
**Mediterranean diet**									
Using olive oil as the principal source of fat for cooking	1	223	94	2.7	0.6,4.8	.09	1.1	−2.4, 4.5	.54
	2	166	93	1.6	−1.1, 4.3	.26			
Having ≥4 tbsp (54 g) of olive oil per day (eg, used for frying, in salads, meals eaten from home)	1	77	33	4.0	−2.0, 10.0	.15	0.0	−9.6, 9.7	.99
	2	77	43	4.0	−3.6, 11.5	.62			
Having 2 or more servings of vegetables per day	1	100	42	10.1	4.0, 16.3	.01	0.4	−9.5, 10.3	.94
	2	66	37	9.8	2.0, 17.5	.01			
Having 3 or more pieces of fruit per day	1	102	43	8.6	2.8, 14.4	.005	9.9	0.6, 19.3	.04
	2	78	44	−1.3	−8.7, 6.1	.48			
Having 1 serving of red meat or sausage per day	1	213	90	6.3	2.2, 10.4	.47	6.6	−0.1, 13.3	.05
	2	134	75	−0.3	−5.5, 4.9	.08			
Having 1 serving of animal fat per day	1	212	90	2.8	−0.2, 5.8	.06	0.6	−4.3, 5.5	.81
	2	166	93	2.2	−1.6, 6.0	.78			
Having 1 cup (100 mL) of sugar-sweetened beverages per day	1	214	91	3.9	0.2, 7.6	.66	1.2	−4.9, 7.3	.70
	2	142	79	2.7	−2.1, 7.4	.03			
Having ≥7 servings of red wine per week	1	43	18	−0.5	−4.3, 3.3	1.0	2.5	−3.7, 8.6	.43
	2	36	20	−2.9	−7.8, 1.9	.17			

		Having ≥3 servings of legumes per week	1	48	20	−2.3	−7.0, 2.4	.71	−0.2	−7.8, 7.5	.97
			2	50	28	−2.2	−8.2, 3.8	.29			
		Having ≥3 servings of fish per week	1	104	44	3.1	−2.7, 9.0	.89	−0.7	−10.3, 8.8	.88
			2	59	33	3.9	−3.6,11.3	.08			
		Having <2 commercial pastries per week	1	123	52	10.5	4.8, 16.2	.003	9.7	0.5, 18.9	.04
			2	83	46	0.8	−6.4, 8.1	.63			
		Having ≥3 servings of nuts per week	1	91	39	−2.4	−8.1, 3.2	.346	−1.1	−10.2, 8.0	.81
			2	58	32	−1.3	−8.5, 5.8	1.0			
		Preferring white meat over red meat	1	163	69	9.6	4.4, 14.7	.005	5.0	−3.4, 13.3	.24
			2	119	66	4.6	−1.9, 11.1	.16			
		Having ≥2 servings per week of a dish with a traditional sauce of tomatoes, garlic, onion, or leeks sautéed in olive oil	1	125	53	1.5	−4.5, 7.4	.62	−2.1	−11.8, 7.5	.66
			2	95	53	3.6	−4.0, 11.2	.49			
		Score for adherence to Mediterranean diet (mean±SD)	1	7.79	2.04	0.53	0.32, 0.74	<.001	0.25	−0.09, .58	.15
			2	7.42	2.18	0.29	0.02, 0.55	.03			
		Study participants with a total score ≥9 points (n %)	1	85	36	11.3	5.3, 17.4	.005	5.8	−3.9, 15.6	.24
			2	57	32	5.5	−2.2, 13.2	.10			
