# Supplementary material for: Long-Term Effectiveness of a Smartphone App for Improving Healthy Lifestyles in General Population in Primary Care: Randomized Controlled Trial (Evident II Study)
Source: JMIR Mhealth Uhealth. 2018 Apr 27;6(4):e107. doi: 10.2196/mhealth.9218 (PMC5948409; doi:10.2196/mhealth.9218)
Supplement: Multimedia Appendix 7 [file mhealth_v6i4e107_app7.pdf]

Table S1. Adherence to app by Stage of change by Prochaska and Diclemente model

**Physical activity stage of change**		**N**	**Mean**	**95% CI**		**P**
Days app used	Precontemplation	57	51.6	42.5	60.8	0.087
	Contemplation	28	58.7	46.2	71.2	
	Preparation	58	49.9	40.1	59.6	
	Action	30	69.3	51.5	87.1	
	Maintenance	237	60.4	55.7	65.2	
	Total	410	58.2	54.6	61.9	
**Dietary habits stage of change**		**N**	**Mean**	**95% CI**		**P**
Days app used	Precontemplation	34	43.9	30.4	57.4	**0.023**
	Contemplation	26	44.6	30.4	58.8	
	Preparation	59	**61.2**	50.8	71.7	
	Action	18	49.0	26.0	72.0	
	Maintenance	275	**61.0**	56.7	65.2	
	Total	412	58.0	54.4	61.7	

Table S2. Changes in physical activity at 12 month by Stage of change by Prochaska and Diclemente model

Acceleromety		N	Mean	95% CI		P
Step / day	Precontemplation	43	-110.9	-966.6	744.7	0.226
	Contemplation	21	-1343.7	-2574.7	-112.7	
	Preparation	43	-1708.6	-2811.9	-605.3	
	Action	26	-620.2	-2066.0	825.6	
	Maintenance	198	-1069.0	-1544.0	-593.9	
Counts minute/week	Precontemplation	43	3.3	-10.6	17.2	0.190
	Contemplation	21	-3.7	-18.1	10.6	
	Preparation	43	-17.2	-27.8	-6.6	
	Action	26	-21.6	-41.1	-2.0	
	Maintenance	198	-15.4	-23.7	-7.1	
Sedentary minute /week	Precontemplation	43	130.1	6.9	253.3	0.839
	Contemplation	21	202.9	-23.1	428.9	
	Preparation	43	219.5	48.7	390.2	
	Action	26	92.7	-129.5	315.0	
	Maintenance	198	162.7	94.3	231.1	
Light minute /week	Precontemplation	43	-121.0	-221.1	-20.8	0.952
	Contemplation	21	-131.3	-327.9	65.3	
	Preparation	43	-144.5	-282.6	-6.4	
	Action	26	-86.1	-241.9	69.6	
	Maintenance	198	-99.3	-152.3	-46.2	
Moderate minute /week	Precontemplation	43	-7.4	-53.7	38.9	0.198
	Contemplation	21	-66.6	-138.6	5.4	
	Preparation	43	-69.7	-117.9	-21.5	
	Action	26	6.2	-74.0	86.4	
	Maintenance	198	-61.4	-89.2	-33.6	
Vigorous very v. minute /week	Precontemplation	43	-1.7	-8.4	5.0	0.607
	Contemplation	21	-5.0	-9.7	-0.3	
	Preparation	43	-5.3	-10.9	0.3	
	Action	26	-12.8	-31.9	6.3	
	Maintenance	198	-2.0	-7.2	3.2	
Total MVPA minute /week	Precontemplation	43	-9.2	-53.5	35.1	0.274
	Contemplation	21	-71.9	-145.6	1.8	
	Preparation	43	-75.2	-126.3	-24.0	
	Action	26	-8.5	-91.0	74.0	
	Maintenance	198	-64.1	-92.7	-35.6	
METS/ minute /week	Precontemplation	43	-39.5	-213.9	134.9	0.409
	Contemplation	21	-296.3	-595.4	2.7	
	Preparation	43	-310.1	-520.8	-99.3	
	Action	25	-109.0	-445.2	227.3	
	Maintenance	197	-256.1	-374.5	-137.6	

7 day PAR		N	Mean	95% CI		P
Total minutes moderate activity	Precontemplation	47	27.4	-23.2	78.0	0.287
	Contemplation	26	16.0	-8.2	40.1	
	Preparation	46	67.4	8.0	126.8	
	Action	26	-45.0	-159.2	69.1	
	Maintenance	202	-13.9	-55.3	27.5	
Total minutes moderate activity in leisure time	Precontemplation	47	22.0	-22.8	66.7	0.112
	Contemplation	26	16.0	-8.2	40.1	
	Preparation	46	94.6	8.5	180.6	
	Action	26	-38.1	-128.4	52.2	
	Maintenance	202	2.0	-31.9	35.9	
Total minutes vigorous/very vigorous activity	Precontemplation	47	-1.9	-5.8	1.9	0.092
	Contemplation	26	5.4	-5.7	16.5	
	Preparation	46	13.7	-6.8	34.2	
	Action	26	-10.4	-32.5	11.7	
	Maintenance	202	-24.9	-41.5	-8.2	
Total minutes vigorous/very vigorous activity in leisure time	Precontemplation	47	0.0	0.0	0.0	0.091
	Contemplation	26	5.4	-5.7	16.5	
	Preparation	46	13.7	-6.8	34.2	
	Action	26	-10.4	-32.5	11.7	
	Maintenance	202	-23.9	-40.3	-7.5	
Total minutes moderate vigorous/very vigorous activity	Precontemplation	47	25.5	-23.5	74.4	0.065
	Contemplation	26	21.3	-4.7	47.4	
	Preparation	46	81.1	20.7	141.5	
	Action	26	-55.4	-171.0	60.1	
	Maintenance	202	-38.7	-83.5	6.1	
**Total minutes moderate vigorous/very v. activity in leisure time**	Precontemplation	47	22.0	-22.8	66.7	**0.011**
	Contemplation	26	21.3	-4.7	47.4	
	Preparation	46	**108.3**	22.0	194.5	
	Action	26	-48.5	-140.7	43.7	
	Maintenance	202	-21.9	-56.5	12.7	
**METS minute/week**	Precontemplation	47	85.3	-103.1	273.7	**0.022**
	Contemplation	26	117.7	-25.4	260.8	
	Preparation	46	**380.9**	106.6	655.1	
	Action	26	-283.2	-757.9	191.5	
	Maintenance	202	-281.3	-504.2	-58.4	
**METS minute/week in leisure time**	Precontemplation	47	82.7	-96.9	262.3	**0.002**
	Contemplation	26	117.7	-25.4	260.8	
	Preparation	46	**489.6**	122.9	856.2	
	Action	26	-255.5	-640.8	129.7	
	Maintenance	202	-209.0	-381.7	-36.4	

Table S3. Changes in Mediterranean diet score at 12 month by Stage of change by Prochaska and Diclemente model

Mediterranean diet		N	Mean	95% CI		P
Mediterranean diet score	Precontemplation	26	0.54	-0.19	1.26	0.817
	Contemplation	21	0.38	-0.48	1.24	
	Preparation	50	0.66	0.08	1.24	
	Action	16	0.06	-0.62	0.75	
	Maintenance	236	0.42	0.18	0.65	
1.Using olive oil as the principal source of fat for cooking	Precontemplation	26	0.04	-0.04	0.12	0.304
	Contemplation	21	0.05	-0.05	0.15	
	Preparation	50	0.00	-0.08	0.08	
	Action	16	0.13	-0.06	0.31	
	Maintenance	236	0.02	-0.01	0.04	
2.≥4 T (1 T=13.5 g) of olive oil/d (eg. used in frying. salads. meals eaten away from home)	Precontemplation	26	0.23	0.02	0.44	0.068
	Contemplation	21	-0.05	-0.35	0.26	
	Preparation	50	-0.10	-0.25	0.05	
	Action	16	-0.06	-0.20	0.07	
	Maintenance	235	0.06	0.00	0.13	
3.2 or more servings of vegetables/d	Precontemplation	26	0.12	-0.09	0.32	0.997
	Contemplation	20	0.10	-0.04	0.24	
	Preparation	50	0.12	-0.02	0.26	
	Action	16	0.06	-0.17	0.30	
	Maintenance	235	0.10	0.03	0.18	
4.3 or more pieces of fruit/d	Precontemplation	26	0.19	0.03	0.35	0.154
	Contemplation	21	0.14	-0.12	0.40	
	Preparation	50	0.04	-0.12	0.20	
	Action	15	-0.20	-0.63	0.23	
	Maintenance	236	0.04	-0.02	0.10	
5. 1 serving of red meat or sausages/d	Precontemplation	26	0.00	-0.20	0.20	0.377
	Contemplation	21	0.19	0.01	0.37	
	Preparation	50	0.04	-0.09	0.17	
	Action	16	-0.06	-0.20	0.07	
	Maintenance	234	0.03	-0.02	0.08	
6. 1 serving of animal fat/d	Precontemplation	26	0.08	-0.08	0.24	0.196
	Contemplation	21	-0.10	-0.29	0.10	
	Preparation	50	0.02	-0.07	0.11	
	Action	16	0.13	-0.06	0.31	
	Maintenance	236	0.03	-0.01	0.06	
7. 1 cup (1 cup=100 mL) of sugar-sweetened beverages/d	Precontemplation	26	0.00	-0.11	0.11	0.261
	Contemplation	21	0.10	-0.19	0.38	
	Preparation	50	-0.02	-0.11	0.07	
	Action	16	0.19	-0.03	0.40	
	Maintenance	235	0.03	-0.01	0.07	
8. ≥7 servings of red wine/week	Precontemplation	26	-0.04	-0.12	0.04	0.922
	Contemplation	21	0.00	-0.14	0.14	
	Preparation	50	0.02	-0.05	0.09	

	Action	16	0.00	-0.19	0.19	
	Maintenance	235	-0.02	-0.06	0.02	
9. ≥3 servings of legumes/week	Precontemplation	25	-0.08	-0.28	0.12	0.630
	Contemplation	21	0.05	-0.05	0.15	
	Preparation	50	0.04	-0.07	0.15	
	Action	16	0.00	-0.19	0.19	
	Maintenance	236	-0.04	-0.09	0.02	
10. ≥3 servings of fish/week	Precontemplation	26	-0.04	-0.18	0.10	0.443
	Contemplation	21	0.10	-0.19	0.38	
	Preparation	50	0.14	0.00	0.28	
	Action	16	-0.06	-0.42	0.30	
	Maintenance	236	0.02	-0.04	0.09	
11. <2 commercial pastries/week	Precontemplation	26	0.04	-0.10	0.18	0.112
	Contemplation	21	-0.10	-0.34	0.15	
	Preparation	50	0.08	-0.05	0.21	
	Action	16	-0.19	-0.54	0.16	
	Maintenance	236	0.10	0.03	0.16	
12. ≥3 servings of nuts/week	Precontemplation	25	-0.08	-0.28	0.12	0.926
	Contemplation	21	0.05	-0.22	0.32	
	Preparation	50	0.00	-0.15	0.15	
	Action	16	0.00	-0.28	0.28	
	Maintenance	235	-0.03	-0.09	0.04	
13. Preferring white meat over red meat?	Precontemplation	26	0.04	-0.20	0.28	0.466
	Contemplation	21	0.00	-0.20	0.20	
	Preparation	50	0.18	0.02	0.34	
	Action	16	0.00	-0.19	0.19	
	Maintenance	236	0.07	0.02	0.13	
14. ≥2 servings/week of a dish with a traditional sauce of tomatoes. garlic. onion. or leeks sautéed in olive oil	Precontemplation	26	0.08	-0.08	0.24	0.395
	Contemplation	21	-0.14	-0.44	0.16	
	Preparation	50	0.10	-0.06	0.26	
	Action	16	0.13	-0.06	0.31	
	Maintenance	236	0.01	-0.05	0.08	
